# Digital Health Interventions for Depression and Anxiety in Low- and Middle-Income Countries: Rapid Scoping Review

**DOI:** 10.2196/68296

**Published:** 2025-08-22

**Authors:** Leena W Chau, Raymond W Lam, Harry Minas, Kanna Hayashi, Vu Cong Nguyen, John O'Neil

**Affiliations:** 1 Faculty of Health Sciences Simon Fraser University Burnaby, BC Canada; 2 Department of Psychiatry Faculty of Medicine University of British Columbia Vancouver, BC Canada; 3 School of Population and Global Health The University of Melbourne Melbourne Australia; 4 Institute of Population, Health and Development Hanoi Vietnam

**Keywords:** digital health, mobile health, mHealth, mental health, depression, anxiety, low- and middle-income countries, LMICs, fidelity, usability, cultural adaptation, COVID-19, implementation science

## Abstract

**Background:**

Low- and middle-income countries (LMICs), which bear a larger proportion of the global mental illness burden, have been disproportionately impacted by the COVID-19 pandemic due to preexisting mental health care system deficiencies. The pandemic has also led to a considerable increase in care delivered through digital mental health interventions (DMHIs), many of which have been adapted from in-person formats. Thus, there is a need to examine their fidelity to the original format along with issues regarding usability and other challenges to and facilitators of their uptake in LMICs. As most DMHIs have been developed in high-income countries, examining their cultural adaptation to LMIC settings is also critical.

**Objective:**

The purpose of this research was to conduct a rapid scoping review of the available evidence on DMHIs for depression and anxiety, two of the most common mental disorders, in LMICs.

**Methods:**

A rapid scoping review was conducted following the PRISMA-ScR (Preferred Reporting Items for Systematic Reviews and Meta-Analyses extension for Scoping Reviews) and processes for rapid reviews by Tricco et al. The PubMed and PsycINFO databases were searched for records published between January 2020 (when COVID-19 was declared a public health emergency) and January 2025 using a search strategy developed in consultation with a liaison librarian. The pandemic accelerated the development and application of DMHIs, and this time frame was used to capture the recent literature that may have incorporated new methods of application. The search strategy was developed across three domains: (1) digital health interventions, (2) depression or anxiety, and (3) LMICs. Data were charted from the final records according to (1) intervention type; (2) discussions on fidelity, usability, and cultural adaptation; and (3) challenges to and facilitators of their uptake in LMICs.

**Results:**

A total of 80 records were included in the final analysis, with reasons for exclusion (eg, focused on mental health in general, not being a DMHI, or not focused on LMICs) reported. Six DMHI platforms were identified: (1) mobile app, (2) the web, (3) virtual reality, (4) videoconferencing, (5) telemedicine, and (6) social media. Less than half of the records referenced fidelity (16/80, 20%), usability (29/80, 36%), and cultural adaptation (31/80, 39%). Challenges pertained to the technological system, engagement issues, structural barriers, and concerns regarding privacy and confidentiality. Facilitators included widespread mobile phone use, built-in supervision and training features, and convenience.

**Conclusions:**

Despite the opportunities that DMHIs offer for reducing the mental health treatment gap, further work examining and improving their fidelity, usability, and cultural adaptation is required. In addition, various challenges to the uptake of DMHIs in LMICs, including contextual issues, structural barriers, and privacy concerns, must be mitigated to avoid contributing further to the digital divide.

## Introduction

### Background

Mental illnesses are a leading contributor to the global burden of disease [1]. The COVID-19 pandemic has contributed to further deteriorations in population mental health and exposed critical and persisting gaps in mental health care [2,3]. The Global Burden of Disease 2020 study estimated that the COVID-19 pandemic has led to increases of 28% in major depressive disorders and 26% in anxiety disorders [4], 2 of the most common mental disorders [5]. Depression and anxiety disorders are associated with profound disease and socioeconomic burden to individuals and their relatives [6]. Low- and middle-income countries (LMICs; countries with a gross national income per capita of ≤US $14,005 [7]), which bear a larger proportion of the global mental illness burden [8], have been disproportionately impacted due to preexisting mental health care system deficiencies [8,9]. Increased mental health issues have, in turn, placed additional substantial pressures on strained mental health care systems [2]. This is particularly concerning in LMICs, where 75% of people lacked access to mental health care before the COVID-19 pandemic [10]. Persistent and growing barriers to treatment in LMICs include low mental health awareness, stigma that impacts help seeking, a shortage and maldistribution of mental health specialists, and care that is concentrated in specialized and tertiary settings with little to nonexisting care at the community level [11,12].

Worldwide, the pandemic has also impacted the delivery of mental health care, leading to a considerable increase in care delivered through digital health interventions to address the widespread reduction of in-person care imposed by public health constraints. These include interventions delivered via telephone, videoconferencing, messaging, web-based programs, and smartphone or other mobile device apps [13], collectively referred to herein as digital mental health interventions (DMHIs). Many of these DMHIs target depression or anxiety. For example, there has been a considerable increase in smartphone apps since the onset of COVID-19. Estimates indicate that >10,000 mental health–related apps are available [14], with most focused on depression and anxiety [6,15]. Digital interventions for depression and anxiety can help with health promotion and literacy at the population level, prevention by targeting high-risk individuals or subpopulations, and treatment and preventive services for people with mental health conditions [16].

Active components of these DMHIs are often based on evidence-based psychological interventions developed for in-person delivery [17]. While it is critical that the interventions are implemented with high fidelity, there is limited evidence on which considerations are most relevant and what should be included in guidance for fidelity of digital adaptations from evidence-based in-person formats. There is a need to evaluate if and how the theoretical and technical components are applied in the new modus or can be enhanced by drawing on the unique interactive features of a digital format (eg, videos and animations) [18]. There has also been limited attention paid to methods for adaptation and evaluations of adaptations across contexts to ensure cultural appropriateness for relevance; applicability; and, ultimately, effectiveness [19-21], particularly for the adaptation of digital interventions [22]. Cultural adaptation can be defined as “the process of systematically modifying an evidence-based intervention to be congruent with the language, values, beliefs, and context corresponding to clients’ cultural backgrounds” [23].

While DMHIs have been used for 2 decades, their development and uptake has been greatly accelerated by the COVID-19 pandemic and hold promise as a feasible way to address the growing mental health needs of populations amidst the persistent and growing mental health treatment gap [12]. This might have been a “black swan” moment as described by Wind et al [24], acting as a catalyst in the marked shift from in-person mental health care provision to digital health. Challenges that have slowed their implementation into routine care include a lack of acceptance by mental health professionals, who may be concerned about threats to the therapeutic alliance, which they believe can only be established in person [25,26]. Other challenges include digital and language literacy [27]; data privacy and security concerns [28], especially as LMICs have seen an increasing number of cyberattacks [29]; and structural issues such as underdeveloped technological infrastructure [12]. There is a need to ensure that all those who need digital mental health care, especially the traditionally underserved groups such as those who live in rural and remote areas, with low income, and from culturally diverse communities, have the digital literacy and opportunity to engage with DMHIs to reduce the digital divide [2]. Another key concern for implementation is usability, the extent to which a product can be used easily, efficiently, and with satisfaction to achieve specified goals [30]. Usability has been identified as a prerequisite for the success of mobile apps [31]. Conversely, poor usability is a primary reason for low user engagement, limiting clinical utility and resulting in the failed adoption of DMHIs [32,33].

### Rationale and Objectives of This Review

Active components of these DMHIs are often adapted from in-person formats [17], most commonly developed and tested in high-income countries [12]. Despite this, there is a paucity of evidence on their fidelity to the original format and limited examination of the cultural adaptation process. Furthermore, although there has been increasing research on DMHIs from LMICs [34,35], where >85% of the global population lives [36], most DMHIs have been developed and studied in high-income countries [12,37], leading to potential biases and limiting generalizability. Knowledge gathered from LMICs will be critical for these countries. Thus, the purpose of this research was to conduct a rapid scoping review of the available evidence on digital health interventions for depression and anxiety, the 2 most common mental disorders, in LMICs.

The specific objectives of this rapid scoping review were to: (1) identify the types of digital health interventions for depression and anxiety being used and studied in LMICs; (2) explore their fidelity, usability, and cultural adaptation to a digital format in LMICs; and (3) examine the challenges to and facilitators of their uptake in LMICs.

This rapid scoping review contributes important evidence on the growing adaptation, use of, and challenges to DMHI applications for mental health in the context of the constantly changing landscape of the COVID-19 pandemic and beyond. While some scoping reviews have focused on digital health interventions for mental health in general [38-40] and others have incorporated a focus on LMICs [11], to our knowledge, this is the first review that focuses on digital health interventions in LMICs specifically for depression and anxiety and includes an in-depth review of their fidelity, usability, and cultural adaptation.

## Methods

### Data Sources and Search Strategy

Considering the need for a timely review, a rapid scoping review was conducted, and a protocol was not registered. We used the following definition of a rapid scoping review as proposed by Tricco et al [41]: “a type of knowledge synthesis in which components of the systematic review process are simplified or omitted to produce information in a short period of time.” Furthermore, scoping reviews seek to provide a broad mapping overview of the extent, range, and nature of the existing evidence base, focusing on breadth regardless of quality [42-44]. For these reasons, appraisal of the methodological quality of the individual studies was not conducted. This rapid scoping review was conducted following the PRISMA-ScR (Preferred Reporting Items for Systematic Reviews and Meta-Analyses extension for Scoping Reviews) and using a search strategy developed in consultation with a liaison librarian at Simon Fraser University. Processes for rapid reviews as outlined by Tricco et al [45] were followed.

Two databases were searched (PubMed and PsycINFO) following common search methodologies for rapid reviews, which advise the search of at least one electronic database (PRISMA [Preferred Reporting Items for Systematic Reviews and Meta-Analyses] standards [46]), with most published rapid reviews using ≥2 [45]. The PubMed and PsycINFO databases were searched for records published between January 2020, when COVID-19 was declared a public health emergency of international concern by the World Health Organization (WHO; declared a pandemic on March 11, 2020), and January 2025. The COVID-19 pandemic accelerated the development of DMHIs, and a rapid review within this time frame was conducted to capture the recent literature that may have incorporated new methods of development and application.

The search strategy was developed across the following three domains: (1) digital health interventions, (2) depression or anxiety, and (3) LMICs. While we were interested in articles related to the COVID-19 pandemic, we made the decision not to include COVID-19 as a search domain as we were interested not only in DMHIs developed to respond specifically to COVID-19–related anxiety and depression but also in DMHIs developed for anxiety and depression in general during the search time frame. Instead, the search included the aforementioned 3 domains and the time frame of the COVID-19 pandemic (2020-2025). A combination of search terms was applied for the automatic search of publications in the aforementioned databases: (“digital” OR “technology” OR “e-health” OR “telemedicine” OR “internet” OR “online”) AND (“depress*” OR “anxi*” OR “mood disorders” OR “anxiety disorders”) AND (“low and middle income countries” OR “developing countries”). Searches included both subject headings and keywords. Search terms for LMICs were taken from the University of Alberta’s Health Sciences Search Filters resource [47]. Multimedia Appendix 1 provides the full search strategy for PubMed, and Multimedia Appendix 2 provides the full search strategy for PsycINFO.

### Screening Strategy and Inclusion and Exclusion Criteria

Records identified in the database search process were screened using titles and abstracts by one researcher (the first author, LWC). Records were selected for full-text review if they: (1) reported on quantitative or qualitative evidence related to DMHIs for depression and anxiety; (2) were from LMICs; (3) were written in the English language; (4) were published between January 1, 2020, and January 31, 2025; and (5) had the full text available. In addition to original research articles, other types of articles (eg, protocol papers and scoping and systematic reviews) were included if they provided information on DMHIs being used in LMICs and included depression or anxiety as a primary focus. We included all articles on depression and anxiety regardless of other conditions or specific patient groups (eg, pregnancy or cancer) as long as the primary outcome included either depression or anxiety.

We excluded articles that did not include depression or anxiety as a primary focus. Any articles that presented data from multiple countries (eg, literature reviews) were included if data from LMICs and non-LMICs were disaggregated and reported separately; in these instances, only data related to LMICs were reported in this review. Duplicates were removed across the 2 databases. Records included for full-text review were cross-checked for relevance and inclusion by an additional researcher (senior author JO).

### Data Extraction and Synthesis

Data were extracted from the records that were eligible through full-text review and charted into 2 tables according to the following headings: author, year of publication, participant characteristics (study location and study population), intervention characteristics (platform and techniques and functions), and study characteristics (design, mental health conditions, and outcomes) in the first table and discussions on fidelity (either in relation to the original in-person or paper-based format or ensuring adherence by lay health workers to the intervention protocol), usability (both quantitative and qualitative explorations), cultural adaptation (of DMHIs to the local cultural context), and challenges to and facilitators of the interventions’ uptake in LMICs in the second table. Findings across the discussions are presented using numerical counts accompanied by descriptive text. See Multimedia Appendix 3 for the PRISMA-ScR checklist.

## Results

### Article Selection

**Figure 1:**
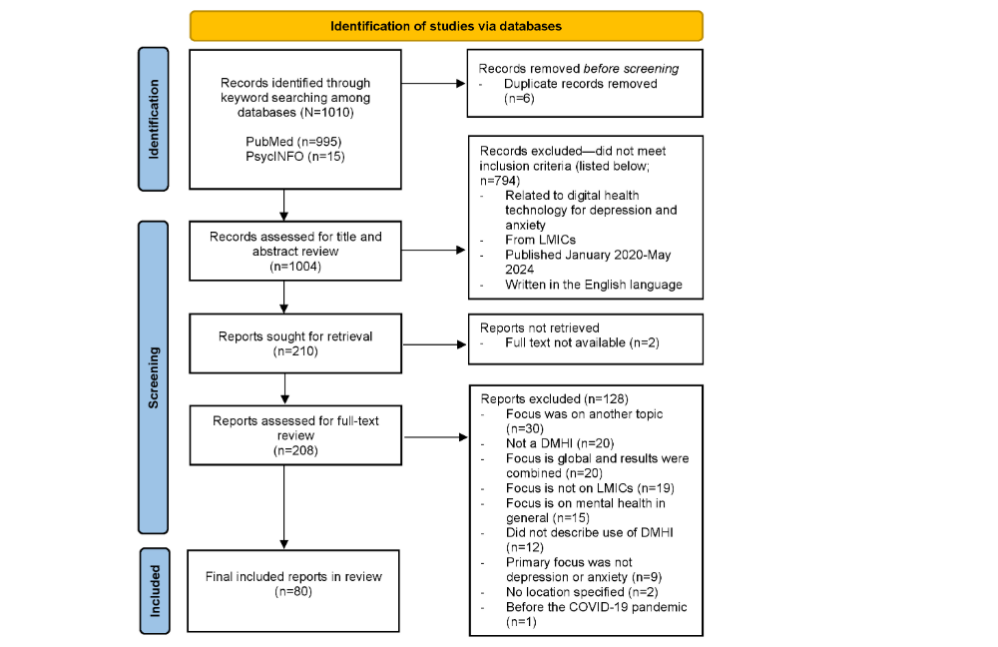
PRISMA (Preferred Reporting Items for Systematic Reviews and Meta-Analyses) flowchart for this rapid scoping review. DMHI: digital mental health intervention; LMIC: low- and middle-income country.

The searches of PubMed and PsycINFO were conducted on March 13, 2025. Figure 1 shows the PRISMA flowchart for the full record selection process. A total of 1010 records were returned, and after the removal of 6 (0.59%) duplicates, 1004 (99.41%) were included for screening. After performing a title and abstract review, 20.92% (210/1004) of the articles were included for full-text review based on our inclusion criteria. A total of 79.08% (794/1004) of the articles, which did not meet the inclusion criteria, were excluded from full-text review. Of the 210 articles included for full-text review, 2 (1%) were not retrieved as the full text was not available.

After completing the full-text review of the 208 available articles, a total of 80 (38.5%) records were included in the final analysis. This included 80% (64/80) original research articles, 15% (12/80) protocol papers, and 5% (4/80) literature reviews (1/4, 25% scoping reviews [48] and 3/4, 75% systematic reviews [49-51]). Reasons for exclusion of articles at full-text review that did not meet the inclusion criteria (128/280, 45.7%) were recorded and reported in Multimedia Appendix 4. The most common reasons for exclusion were focus on another topic (eg, app use in general), lack of a DMHI (eg, therapists using video or telephone to deliver regular counseling without an accompanying app or online platform), results from reviews (global focused) combined without data from LMICs being reported separately, focus on mental health in general, and no description of the use of a DMHI. A quality appraisal was not conducted considering the nature of this rapid scoping review, which did not focus on an examination of the effectiveness of the DMHIs, along with the emergent nature of the literature. The examinations of fidelity and usability were reviewed and described independent of whether there was an exploration of intervention effectiveness and, if there was, whether the intervention was effective.

### Study Characteristics

Table 1 presents the main characteristics of the studies included in the rapid scoping review. Six main DMHI platforms were identified: (1) mobile app (software app used on a mobile device such as a smartphone or tablet), (2) web-based program (intervention delivered on the web through an internet-connected device), (3) virtual reality (VR; involved the immersion of the user in simulated and controlled computer environments [52]), (4) videoconferencing (intervention delivered via video through an online platform; eg, Google Meet and Zoom), (5) telemedicine (intervention delivered via phone calls or text through an instant messaging app [eg, WhatsApp] that went beyond the usual psychoeducation delivered by therapists by incorporating additional components such as educational videos and relaxation music), and (6) social media–based interventions (delivered through a social media platform; eg, Facebook).

**Table 1:** Summary of the included articles (participant characteristics, intervention characteristics, and study characteristics).

Study	Participant characteristics			Intervention characteristics			Study characteristics	
	Location	Population	Platform		Techniques and functions	Design		Mental health condition, with results where applicable
Aalaei et al [53], 2021	Iran	Hospitalized patients who tested positive for COVID-19 at Imam Reza Hospital in Mashhad (N=112)	Mobile phone app		Mobile-based psychological counseling and health status monitoring	Protocol RCT^a^—2 parallel groups		Depression and anxiety
Abbasalizadeh et al [54], 2024	Iran	ICU^b^ nurses from 2 Tehran hospitals (N=60)	Android smartphone app		Resilience training developed based on the microlearning educational method	RCT—single blinded		Anxiety and stress; significant reductions in stress from 10.77 on DASS-21^c^ (SD 3.33) to 9.00 (SD 1.66; *P*=.001) and in anxiety from 9.43 on DASS-21^s^ (SD 3.35) to 7.93 (SD 0.98; *P*<.001)
Abi Ramia et al [55], 2023	Lebanon	Participants from 2 previous RCTs that demonstrated effectiveness of the intervention, staff members, and external stakeholders	Digital intervention (SbS^d^) delivered through an app or website		Guided self-help intervention	Qualitative study involving key informant interviews		Depression; high level of acceptability of SbS among Lebanese and Syrian participants
Akbari et al [56], 2022	Iran	Home-isolated patients with COVID-19 visiting Sina Hospital, Hamadan (N=60)	WhatsApp messages and instructional videos		Virtual training of relaxation techniques via WhatsApp messages and instructional videos in the form of 8 educational messages, delivered online	RCT—2 group, parallel		Anxiety; relaxation techniques were effective in reducing anxiety among patients with COVID-19
Akhtar et al [57], 2024	Pakistan	Community health workers (LHWs^e^; N=44)	Mobile app (mPareshan)		3 segments: tracking, counseling, and referral; LHWs facilitated psychoeducation through audio and video features	Qualitative study involving focus groups and interviews		Depression
Alavi and Hirji [58], 2020	Iran	General population with a Generalized Anxiety Disorder diagnosis from a psychiatrist (N=152)	Computer-based intervention delivered via email		Weekly (×12) e-CBT^f^ intervention modules and homework assignments delivered via email, with structured support from trained clinicians	RCT		Anxiety (generalized); a mixed model ANOVA showed a significant difference between the groups and BAI^g^ evaluation periods (*F*_3,57_=101.5, *P*<.001).
Al-Badiri et al [59], 2024	Iraq	Registered nurses at Al-Alhamzeh General Hospital (N=72)	iMBSR^h^		Guided mindfulness exercises, reflective discussions, and educational materials delivered over 8 sessions	Semiexperiment with a pretest-posttest design		Depression; a nonsignificant decrease in PHQ-15^i^ mean scores in the iMBSR group from 9.19 (SD 3.07) to 7.58 (SD 3.61; *P*=.17)
Alishahi et al [60], 2025	Iran	Patients undergoing hemodialysis at Montaserieh Hospital (N=72)	Mobile app		Recreational therapy mobile app comprising programs such as music, comedy movies, exercise routines, and educational question-and-answer games	RCT		Depression; the intervention group showed a significant decrease in depression scores compared to the control group (mean BDI-II^j^ score reduction in the intervention group 10.3, SD 4.1; mean score reduction in the control group 4.6, SD 3.8; *P*<.001)
Amer et al [61], 2023	Egypt	Adults aged ≥18 y from Mansoura University’s Faculty of Computers and Information (N=5)	Mobile app (Sokoon)		Gamified CBT app focused on 7 intervention skills: relaxation, gratitude, self-compassion, problem-solving, social skills, BA^k^, and cognitive restructuring	Pilot study		Depression and anxiety; anxiety symptoms decreased from the pretest (mean GAD-7^l^ score of 10, SD 0.7) to the posttest (mean 4.2, SD 1.5), and depressive symptoms decreased from a mean PHQ-9^m^ score of 10 (SD 2.05) to a mean of 6 (SD 1.7)
Amer et al [62], 2024	Egypt	Adults aged ≥18 y from Mansoura University’s Faculty of Computers and Information (N=30)	Mobile app (Sokoon)		CBT app that incorporated techniques such as Hexad theory, gamification, and dynamic difficulty adjustment	Single-group pretest-posttest design		Depression and anxiety; positive impact on reducing symptoms of depression and anxiety
Araya et al [63], 2021	Brazil	Patients with depressive symptoms with comorbid hypertension or diabetes (n=880 in Brazil; n=432 in Peru)	Smartphone intervention		Low-intensity smartphone intervention delivered through 18 sessions over 6 wk; based on BA and supported by nurse assistants	2 RCTs; 1 cluster randomized in Brazil and 1 individual-level randomized in Peru		Depression; significantly improved depression symptoms at 3 mo; effect size small in Brazil, and effects were not sustained at 6 mo
Atif et al [64], 2022	Pakistan	Women living in resource-poor rural communities (n=3) and peer delivery agents (n=3)	Tablet- or smartphone-based app (Thinking Healthy Programme)		CBT intervention delivered by a virtual avatar therapist that was operated by a lay peer; the peer and the avatar acted as cotherapists	Usability testing study		Depression (perinatal)
Bhardwaj et al [65], 2025	India	Family caregivers of persons living with dementia	Mobile app		CBT-based psychoeducational intervention developed based on the WHO^n^ iSupport program delivered in 10 weekly sessions; ASHAs^o^ will be trained to deliver the intervention	Protocol community-based cluster RCT		Depression
Bhargav et al [66], 2024	India	Individuals with a COVID-19 positive test from an NABL^p^-approved laboratory who also reported at least a moderate level of stress (a score of ≥14 on the PSS^q^; N=86)	Tele-yoga		Tele-yoga intervention delivered via videoconference supervised by trained yoga therapists	Prospective, multicenter, open-label single-arm study		Depression and anxiety; anxiety improved significantly at wk 4, and the improvements were sustained at wk 16
Bhat et al [67], 2023	India	Women in self-help groups	Tablet app (MITHRA^r^)		MITHRA for depression screening (PHQ-9^m^ and brief intervention [HAP^s^ modules])	Protocol—focus groups		Depression
van Heerden et al [68], 2021	Nepal	Lay counselors caring for adolescent mothers with depression	Platform (StandStrong) comprising the StandStrong counselor mobile app and a cloud-based processing system		App visualized passively collected GPS, proximity, and activity data; lay counselors used the app to discuss mothers’ behavioral patterns and clinical progress over the 5-wk intervention	App development paper		Depression (maternal)
Chand et al [69], 2024	India	Individuals from the Indian Institute of Technology Mandi (N=44)	VR^t^—music		Raga Bhairavi, an Indian classical music, administered via 360° video in a VR environment for 6 d (each session lasting 15 min)	RCT—between-group repeated measures		Depression and anxiety; all DASS-21 subscales significantly improved; a post hoc test showed that the anxiety and depression levels were significantly lower for the treatment group after 6 d of treatment (anxiety: t_21_=5.77, *P*<.001, and Cohen *dz*=1.04; depression: t_21_=5.16, *P*<.001, and Cohen *dz*=1.16)
Chau et al [70], 2023	Vietnam	Adults aged ≥18 y in community-based settings	Smartphone app (Vmood)		Supported self-management based on CBT principles with supportive coaching from a social worker delivered via chat through an app	Protocol RCT—stepped wedge		Depression and anxiety
Cuijpers et al [71], 2022	Lebanon	People with depression residing in Lebanon (N=680)	Internet-connected device (SbS)		5-session depression intervention; provided psychoeducation and training in BA through an illustrated narrative delivered with guidance from nonspecialist helpers	RCT—single blind, 2 arm, pragmatic		Depression; ITT^u^ analyses showed effects on depression (SMD^v^ 0.71, 95% CI 0.45-0.97) and impaired functioning (SMD 0.43, 95% CI 0.21-0.65)
Cuijpers et al [72], 2022	Lebanon	Syrian refugees living in Lebanon (N=569)	Internet-connected device (SbS)		5-session depression intervention; provided psychoeducation and training in BA through an illustrated narrative delivered with guidance from nonspecialist helpers	RCT—single blind, 2 arm, pragmatic		Depression; ITT analyses showed significant treatment effects for both primary outcomes, depression (moderate) and functional impairment (moderate), and significant effects on anxiety
Dambi et al [73], 2022	Zimbabwe	General population (N=176)	Digital intervention delivered via web application or mobile app (Inuka)		A chat-based digital application; participants received 6 problem-solving therapy sessions delivered by lay counselors	Feasibility study—pragmatic quasi-experimental design		CMDs^w^, including depression and anxiety
Doosti et al [74], 2024	Iran	Patients with gynecological cancer referred to educational and therapeutic centers affiliated with the Shahrekord University of Medical Sciences (N=70)	Mobile app		Main menu featured various options (eg, entering clinical data, receiving weekly patient status reports, and virtual consultations with nurses); users could easily interact with the researcher through a message box icon	RCT—2 group, pretest-posttest		Anxiety; state anxiety scores significantly decreased in the intervention group from 51.64 (SD 14.97) to 40.20 (SD 11.70) at the follow-up, and trait anxiety scores in the intervention group decreased significantly from 49.91 (SD 14.96) to 39.82 (SD 10.28), whereas the scores of the control group worsened
Dosani et al [48], 2020	LMICs^x^	Women	Various		Various	Scoping review		Depression (perinatal); 12 articles met the inclusion criteria; results revealed that minimal literature is currently available on the use of mHealth^y^ for perinatal depression in LMICs
Ebrahimi et al [75], 2025	Iran	Patients with ailurophobia referred to the outpatient clinic of Ibn Sina Specialized Hospital and psychiatry and psychology clinics in Mashhad (N=28)	Two interventions: (1) VR and (2) metacognitive sessions		VR sessions with images of cats; metacognitive sessions that evaluated symptoms, recognized cognitive errors, and corrected beliefs	RCT		Anxiety (ailurophobia); statistically significant difference between the average anxiety score of the 2 groups (VR and metacognitive therapy; *P*˂.001); anxiety score was significantly reduced in the VR group
Fu et al [49], 2020	LMICs	Various	mHealth		Various	Systematic review and meta-analysis		22 studies were included; digital psychological interventions, which have been mostly studied with depression and substance misuse, were superior to control conditions, including usual care, and were moderately effective in LMICs
Ganjekar et al [76], 2024	India	Pregnant women from 3 tertiary care hospitals (N=162)	Web-based pregnancy tele-yoga module		Tele-yoga delivered and monitored through an online platform over 4 wk	Multicentric, open-label, exploratory study		Depression and anxiety; anxiety symptoms persisted in 19 (19.78%) women, and depression persisted in 10 (10.41%) women; after the intervention, none of the pregnant women reported severe symptoms
Gautama et al [77], 2024	Indonesia	Patients with cancer undergoing chemotherapy (N=99)	SVR^z^ relaxation intervention		10-min immersive VR experience featuring 360° natural scenery videos with relaxing sounds delivered through YouTube via a smartphone	RCT—prospective, 2 arm		Anxiety; the SVR group reported significantly reduced anxiety (*P*=.01; Cohen *d*=0.50) compared to the control group
Ghanbari et al [78], 2021	Iran	Women with nonmetastatic breast cancer aged 20-60 y (N=82)	Smartphone app (BCSzone) and online support group		Guided self-management psychoeducational intervention delivered through BCSzone along with nurse-assisted online mobile support sessions delivered through WhatsApp over a total of 4 wk	RCT—unblinded		Anxiety; statistically significant differences between the mobile psychoeducation group and controls (*P*<.001); the paired *t* test for mean scores of anxiety showed significant reductions
Gonsalves et al [79], 2022	India	Secondary school students (N=11)	Smartphone app (POD Adventures)		Gamified problem-solving intervention supported by nonspecialist counselors via phone	Parallel, 2-arm, individually randomized controlled pilot trial		Depression and anxiety; no intervention arm participants completed the intervention because participants could not be reached
Green et al [80], 2020	Kenya	Pregnant women and new mothers recruited from public hospitals (N=41)	Tablet or smartphone app (Healthy Moms)		Task-sharing Thinking Healthy program adapted for automated delivery via a mobile phone or tablet; used an existing AI^aa^ system called Tess (Zuri in Kenya) to drive conversations with users	Prepilot study; single-case experimental design		Depression (perinatal); authors estimated that using the alpha version of the app may have led to a 7% improvement in mood
Gupta et al [81], 2022	India	Health care providers from a tertiary care medical center (N=202)	Web based		Two groups: (1) Raja yoga meditation—structured module of Raja yoga for 3 mo taught by a qualified teacher and (2) stress management counseling—dedicated trainer who counseled and taught 7 steps for managing stress for 3 mo	Prospective observational study		Depression and anxiety; practice of Raja yoga meditation for 12 wk resulted in significant decrease in DASS-21 score (−45.00 at baseline and −24.2 at 12 wk; *P*<.001); no significant decrease was observed in the DASS-21 score in the stress management counseling group at 12 wk of follow-up
Iravani et al [82], 2023	Iran	Pregnant women from health centers (N=104)	Telemedicine		Counseling services delivered by a researcher through telephone conversations; SMS text messages; and apps such as WhatsApp, Telegram, and Instagram over a 2-mo period; educational video clips were provided through a mobile app	Quasi-experimental study		Anxiety; health anxiety scores of pregnant women in the control group were significantly higher than those of pregnant women in the experimental group; the mean scores of pregnancy-related anxiety in the control group were significantly higher than those in the intervention group
Jannati et al [83], 2020	Iran	Women aged ≥18 y who attended health care centers in Kerman, Iran (N=75)	Mobile phone app (Happy Mom)		CBT app consisting of 8 lessons conducted over 8 wk; participants followed the story of women with PPD^ab^ and learned how they managed their symptoms, which they could apply to their own lives	Nonblinded parallel-group RCT		Depression (post partum); average Edinburgh Postnatal Depression Scale score after the intervention was 8.18 in the intervention group and 15.05 in the control group, which was statistically significantly different (*P*<.001)
Kanuri et al [84], 2020	India	College students	Online intervention (Mana Maali Digital Anxiety Program)		Culturally and digitally adapted evidence-based CBT intervention delivered over 20 sessions for anxiety	Initial usability, acceptability, and feasibility examination		GAD; results indicated high usability, with the average rating ranking in the top 10% of general usability scores
Kim et al [50], 2023	LMICs	General population	Digital mental health tools		Various	Systematic review and meta-analysis		Depression and anxiety; 80 studies included in the meta-analysis and 96 studies included in the systematic review; digital mental health tools were moderately to highly effective in reducing depression and anxiety symptoms in LMICs
Kola et al [85], 2022	Nigeria	Adolescent mothers (n=17) and maternal health care providers with experience in perinatal depression (n=25) who were participants of a previous clinical trial called EXPONATE^a^^c^	Mobile phone intervention		Design of the EXPONATE intervention, which incorporated the treatment specifications of the WHO mhGAP-IG^ad^; 4 components: parenting skill training, problem-solving, psychoeducation, and use of available social support	Focus groups		Depression (perinatal); focus group discussions showed that 70% of the barriers identified related to the inner setting and the characteristics of individuals in the intervention (eg, clinicians’ knowledge deficit on the care of young mothers with depression and young mothers’ lack of interest in motherhood)
Kusumadewi et al [86], 2025	Indonesia	Medical students from Gadjah Mada University, Yogyakarta (N=66)	Smartphone app		App using asynchronous digital cognitive education divided into 3 parts, including information, therapy (based on Beckian cognitive therapy), and daily journal sections	RCT—nonblinded		Anxiety; significant effect on reducing anxiety scores from the second to the eighth wk
Lahti et al [87], 2020	South Africa and Zambia	Higher education institute academics and PHC^ae^ practitioners in 3 provinces in South Africa and 2 provinces in Zambia	Mobile app		Screening tool to be used in PHC settings	Protocol; mixed methods multisite design		Depression
Logie et al [88], 2021	Uganda	Urban refugee and displaced youths aged 16-24 y	VR and GPM+^af^		VR-only arm: immersive, interactive 15-min VR session that integrated mental health literacy, psychological first aid, and mindful self-compassion; VR and GPM+ intervention: VR intervention and GPM+, (WHO brief psychological intervention) delivered over 5 group sessions by peer navigators	Protocol; 3-arm cluster RCT		Depression
Luitel et al [89], 2023	Nepal	Primary HCWs^ag^ trained in using the WHO mhGAP^ah^ mobile app in Jhapa (N=15)	Smartphone app (WHO e-version of the mhGAP)		The WHO launched the e-version of the mhGAP in 2017; 8 modules to address key priority mental health conditions, including depression	Qualitative		Mental health, including depression; HCWs found the app helpful to verify and confirm their assessment and diagnosis
Maharjan et al [90], 2021	Nepal	Adolescent and young mothers with (n=11) and without (n=27) depression	Smartphone app (StandStrong)		Through a (1) smartphone app and (2) Bluetooth beacon, sensing data were passively collected on mothers’ geographic movement, physical activity, and audio environment and mother-infant proximity	Feasibility study		Depression
Mazhari et al [91], 2022	Iran	Inpatients with COVID-19 at Afzalipour Hospital (N=200)	Web-based eHealth intervention		A questionnaire administered via WhatsApp; after 2 d, the psychiatric resident video called those who completed the questionnaire to assess depression (Hamilton Depression Rating Scale) and anxiety (Hamilton Anxiety Rating Scale)	Cross-sectional descriptive study		Depression and anxiety; this study examined the agreement between the online self-assessment and psychiatric telephone interview among survivors of COVID-19 regarding depression and anxiety; agreement between the online self-assessment and telephone interview was not significant
Moulaei et al [92], 2023	Iran	Patients with depression and anxiety disorders from Kerman University of Medical Sciences	Mobile app		Self-management of depression and anxiety disorders	App development paper		Depression and anxiety
Mukherjee et al [93], 2024	India	Adolescents living in 60 slum clusters spread across New Delhi and Vijayawada	Mobile app		Two components: (1) antistigma campaign and (2) mobile-based self-care app	App development paper		Depression and anxiety
Muniswamy et al [94], 2022	India	Remotely working software professionals in IT firms who were low in physical and mental health (N=160)	Social media (Facebook)–based FIIT^ai^		Modules provided via weekly Facebook messages focused on factors affecting sedentary behavior and mental health delivered over 8 wk; personal telehealth consultations delivered by experts via a chat box	RCT—2 phases, pragmatic		Anxiety and depression; significant difference in the scores for stress and anxiety within and between the groups but only within the groups for depression
Nabunya et al [95], 2024	Uganda	Youths aged 14-17 y who were HIV positive and receiving care and ART^aj^ from one of the participating clinics and living within a family	Mobile intervention (Suubi-Mhealth)		Educational content incorporated recommendations for CBT for ART adherence and depression as applied to HIV medication adherence; content was broken down into 20 modules	Protocol—pilot RCT		Depression
Najafi et al [96], 2023	Iran	Female individuals with RRMS^ak^ recruited through the local MS^a^^l^ association (N=45)	Online visual platforms: Google Meet, Zoom, and Instagram		Online supervised tele-yoga and tele-Pilates intervention sessions delivered 3 times a wk over 8 wk; certified instructors provided supervision	RCT—single blind		Depression; significant improvements were observed in depression (*P*=.001)
Najafi et al [97], 2023	Iran	82 people with either RRMS or SPMS^am^ from the Shiraz University of Medical Sciences (N=82)	Tele-Pilates and tele-yoga training		Online supervised tele-yoga and tele-Pilates intervention sessions delivered 3 times a wk over 8 wk; certified instructors provided supervision	RCT		Depression; significant time × group interactions were observed for mental health (*P*=.05), as well as a decline in depression (*P*=.002); post hoc analysis showed depression improvements for both interventions, but these were not statistically significant (*P*>.05)
Naveen et al [98], 2024	India	HCWs working at AIIMS^a^^n^, Jodhpur on COVID-19 duty (N=90)	Tele-yoga		Module contained loosening practices, breathing practices, Surya Namaskar, Kapalabhati Kriya, pranayama, and relaxation techniques	RCT		Depression and anxiety; no significant difference in depression scores at endline; anxiety scores were significantly lower among the tele-yoga group than among control group participants (*P*<.05) at endline; the effect size was medium (*r*=0.3-0.5), favoring the intervention group
Njoroge et al [99], 2023	Kenya	HCWs from 5 health care facilities	Mobile app		Weighted sensor stream signatures that predicted near-term risk of a major depressive episode and future mood; deployment of a mobile app platform and use of novel analytic approaches (AI and machine learning)	Feasibility study		Depression
Nurbaeti et al [100], 2021	Indonesia	Convenience sample of postpartum mothers	Smartphone app (IMSD^a^^o^)		IMSD app sought to detect PPD symptoms early	App development and RCT		Depression (PPD)
Nwaogu et al [101], 2021	Nigeria	Construction personnel—site supervisors and project managers based on a log collected from the Nigerian Institute of Building (N=62)	DMHI^a^^p^ (various)		Various	Open-ended survey—qualitative technique		Anxiety and depression; DMHIs used by respondents included mobile-based apps (14/24, 58%) and web-based applications (10/24, 42%) for stress, depression, anxiety management, and mental fitness
Ofoegbu et al [102], 2020	Nigeria	Educational technology students of Nigerian universities identified as having depression (N=192)	Internet-delivered intervention (GIAI^a^^q^)		Initial support session via telephone and regular guidance from the therapists (clinical staff of the federal universities) via telephone and the internet over 10 wk	Group-randomized trial		Depression; significant reduction in depression among students in the treatment group when compared to those in the usual care control group
Pasyar et al [103], 2023	Iran	Patients with systemic lupus erythematosus referred to the Hafez clinic affiliated with Shiraz University of Medical Sciences (N=124)	Smartphone-based supportive counseling		Daily supportive counseling using text and multimedia messages for 8 wk delivered via a WhatsApp channel; feedback and questions could be sent to research team members	RCT—pretest-posttest		Anxiety (health); the level of health anxiety (MD^a^^r^ 11.34; *P*<.001) of the experimental group was significantly lower than that of the control group
Patel et al [104], 2021	Fiji	CHNs^a^^s^ (N=48)	mHealth app (ASRaDA^at^)		Guidelines from the CES-D^au^ and the SBQ-R^av^ for suicide were programmed into ASRaDA and provided online	Experimental design		Depression; CHNs took less time to process patient information with more accurate diagnostic and therapeutic decisions compared with the paper-based or no-guideline conditions
Pozuelo et al [105], 2023	South Africa and Uganda	Adolescents in rural South Africa and Uganda (N=160)	Smartphone app (Kuamsha)		App delivered BA using storytelling techniques and game design; each story consisted of 6 modules, with each covering different BA skills; included support via phone calls	App development paper—iterative user-centered agile design		Depression
Putri et al [106], 2022	Indonesia	Participants who used 8 of the most popular Android mHealth platforms	mHealth platforms		Teleconsultation focus	Mysterious shopping approach		Depression
Rabbani et al [107], 2024	Pakistan	LHWs (n=72) and screen-positive participants (residents who were aged ≥18 y; n=366)	Mobile app		Counseling intervention consisting of tracking, counseling, and referral segments delivered by LHWs	Protocol single-arm, pretest-posttest implementation research trial		Depression and anxiety
Ramnath and Suri [108], 2021	India	General population of patients both with and without COVID-19	Smartphone app		Online survey explored the needs of individuals with depression in India, and a systematic evaluation of depression apps currently available investigated user needs	Online survey+systematic evaluation		Depression
Roshid et al [109], 2024	Bangladesh	Informal female caregivers of patients with cancer in Bangladesh	mHealth psychoeducation and BRT^aw^		Used self-determination theory and the BRT; included 6 sessions over 6 mo where recorded videos would be provided face-to-face or via a digital device	Protocol RCT—prospective, open label, 2 arm (1:1)		Anxiety and depression
Safaei et al [110], 2024	Iran	Patients undergoing angiography from 2 hospitals affiliated with Kerman University of Medical Sciences (N=70)	Mobile app		App with 13 sections that addressed various aspects of the patient journey, providing comprehensive support and guidance throughout and after the angiography process	Interventional study		Anxiety; significant decrease in average anxiety scores (*P*<.001)
Saleem et al [111], 2025	Pakistan	LHWs (N=40)	Mobile app (TA-PM+^ax^)		App that guided LHWs to deliver the intervention in a systematic manner; included 5 informational videos	Coproduction and usability study		Depression and anxiety; usability testing results: the mean MAUQ^a^^y^ score was 5.62 in round 1 of usability testing and improved to 5.96 after incorporating LHW feedback in round 2, demonstrating the system’s overall usefulness
Taylor Salisbury et al [112], 2021	Nepal and Nigeria	Primary care workers and patients from approximately 20 PHC clinics in Nepal and 6 PHC clinics in Nigeria	Mobile app (WHO-updated e-mhGAP-IG)		App for diagnosis and management of depression; featured a reference mode for training and a patient mode for assessments	Feasibility study		Depression
Sarpourian et al [52], 2022	Iran	Students at the undergraduate, postgraduate, and PhD levels from the Kerman University of Medical Sciences and Shiraz University of Medical Sciences (N=30)	VR and counseling therapy		Participants observed 4 virtual classroom scenarios in a 30‐min session, which included a virtual classroom and comprised 4 parts	Quasi-experimental study		Anxiety (public speaking); VR and counseling did not affect students’ SA^a^^z^, but they reduced PSA^ba^
Seddigh et al [113], 2023	Iran	Adolescent girls with type 1 diabetes who had a clinical record at Shiraz’s Imam Reza diabetes clinic (N=62)	Yoga therapy via virtual training		Eight 60-min sessions (1 online and 7 offline) of yogic practices held for 8 consecutive wk through Adobe Connect Reader for 90 min	RCT		Depression; the mean of the depression total score was significantly lower in the experimental arm; the reduction in mean change from baseline to the trial end was significantly higher in the experimental arm
Setiawan et al [114], 2025	Indonesia	Patients diagnosed with thalassemia from a general hospital in the Priangan Timur region (N=80)	Mobile app (Cyber Gen)		Genetic counseling delivered through the chat feature on an Android-based mobile app (Cyber Gen) conducted in 3 separate sessions	RCT		Depression and anxiety; depression and anxiety in the intervention group showed significant changes across all observed periods (*P*<.001), with a large effect size (Cohen *d*>0.8)
Shania et al [115], 2023	Indonesia	Adolescents	Mobile app		App included a mood tracker, activity target, and meditation, along with supporting features (educational articles) and early detection features	Mixed methods to design an mHealth intervention		Depression
Shaygan et al [116], 2023	Iran	Patients with COVID-19 in home quarantine (N=72)	Telephone intervention		Videos, audio files, and educational texts regarding mental health, coping strategies, positive thinking, hope, and spiritual well‑being; interventions were provided daily for 14 d	RCT		Anxiety; state‑trait anxiety was significantly lower in the interactive psychoeducational intervention group; the mean score of state anxiety was 34.69 (SD 10.75) in the intervention group and 45.75 (SD 13.01) in the control group after the intervention, with a large effect size
Singla et al [117], 2024	India	Embedded in 3 programs to scale up the HAP; included participants from those programs	Smartphone app (PEERS^bb^)		PEERS app aimed to facilitate remote registering and scheduling patients, collecting patient outcomes, rating therapy quality, and assessing supervision quality among frontline treatment providers delivering BA for depression	Mixed methods		Depression
Sinha et al [118], 2023	United Kingdom, United States, and India	General population	Digital mental health app (Wysa)		Wysa was an AI-enabled, chat-based app aimed at building resilience; it included a conversational agent and various evidence-based intervention tools and techniques, such as CBT and mindfulness	Retrospective observational design		Depression and anxiety
Soltani et al [119], 2024	Iran	Patients with MDD^bc^ referred to Razi Hospital and the psychiatry clinic of Madani Hospital (N=64)	Smartphone app (Yara)		Included a section with 3 tests for the daily report of patients’ sleep quality, suicidal thoughts, and anxiety; content included a daily exercise video; the client received motivational sentences daily and recorded their daily activities	RCT—pretest-posttest design		Depression and associated symptoms
Than et al [120], 2023	Vietnam	Family caregivers of people with dementia living in the community who attended the Department of Geriatrics at Gia Dinh People’s Hospital	Smartphone app		App will include weekly online psychoeducational group sessions hosted on the Zalo app; caregivers will be added to a “Caregiver Support Group” room in Zalo; weekly, the administrator will post 1 topic for discussion	Protocol pilot RCT		Depression and anxiety
Torrey et al [121], 2020	Colombia	Patients from 2 primary care clinics, 1 urban and 1 rural, in a small town (N=2656 screened)	Technology-supported care		Included waiting room kiosks to screen primary care patients, decision support tablets to guide doctors in diagnosis and treatment, and access to digital therapeutics	Descriptive study		Depression; primary care physicians increased the percentage of patients diagnosed as having depression and alcohol use disorder from next to 0% to 17% and 2%, respectively
van der Watt et al [51], 2020	Chile, Denmark, South Africa, and the United States	Various	Various		Remote mood monitoring that included distant support in participants with mood disorders	Systematic review		Depression; distant mood monitoring was effective in improving depression scores but not mania scores; feasibility varied
van Heerden et al [122], 2024	Nepal	Counselors and adolescent mothers from postnatal clinics and immunization camps in Chitwan (N=24)	Mobile app (StandStrong; Sensing Technologies for Maternal Depression Treatment in Low Resource Settings)		For lay counselors offering a 5-session BA intervention (HAP) to visualize the passive sensing data of young mothers with depression for counseling sessions	Proof-of-concept study (single-arm intervention)		PHQ-9 scores at screening averaged 12.13 (SD 2.85); no statistically significant change reported
Vera Cruz Dos Santos et al [123], 2021	Brazil and Peru	Patients of public health care services for hypertension, diabetes, or both conditions in São Paulo, Brazil (n=880), and Lima, Peru (n=442)	Smartphone app (CONEMO^bd^)		mHealth intervention based on BA with minimal support from a nurse or nurse assistant (with weekly supervision from a psychiatrist); included 18 brief, automated mini sessions delivered over a 6-wk period	Protocol—economic evaluation alongside 2 RCTs		Depression
Watanabe et al [124], 2024	Vietnam and Thailand	Nurses working in hospitals in Vietnam and Thailand	Mobile app (ABC Stress Management–COVID-19 version)		7-wk smartphone-based self-guided iCBT^be^ program for nurses; consisted of texts and static visuals of cartoon characters talking about the study topic and guiding participants	RCT—2 arm, parallel group		Depression; in both groups, the average scores of depression at the 3- and 6-mo follow-ups were lower than those at baseline; Cohen *d* values were –0.13 (95% CI –0.26 to –0.02) at the 3-mo follow-up and –0.05 (95% CI –0.17 to –0.02) at the 6-mo follow-up
Weierstall et al [125], 2021	Burundi	Burundian AMISOM^b^^f^ (UN^bg^-approved mission) soldiers (N=463)	Tablet app		Structured clinical interviews (in person) for assessments of clinical symptom profiles by rating symptoms and responses in field research (diagnostic procedure) via tablets	Feasibility study		Depression; findings demonstrated that tablet-supported assessments can provide a useful application of mobile technology in large-scale studies, especially in resource-poor settings
Zamanifard et al [126], 2022	Iran	Children with type 1 diabetes aged 8-12 y who were referred to Imam Reza diabetes clinic in Shiraz (N=40)	Virtual painting therapy		Six 2-h group therapy sessions delivered over 6 wk via WhatsApp video call by the main researcher; children were encouraged to draw anything, those who showed anxiety and depression in their paintings were identified, and reports were sent to their parents for follow-ups	RCT		Depression and anxiety; significant difference between the groups regarding anxiety (*P*=.02) and depression (*P*<.001); however, the difference in self-efficacy was not significant (*P*=.20)
Zuccolo et al [127], 2021	Brazil	Pregnant women aged between 16 and 40 y with depression	Smartphone app (Motherly)		On the basis of 3 concepts to deliver BA: psychoeducation, behavior monitoring, and gaming elements; package of interventions comprised various modules, such as mental health and sleep; included 4 online sessions of brief CBT in 8 wk	Protocol—2-arm RCT		Depression (in pregnant women)

^a^RCT: randomized controlled trial.

^b^ICU: intensive care unit.

^c^DASS-21: Depression, Anxiety, and Stress Scales–21.

^d^SbS: Step-by-Step.

^e^LHW: lady health worker.

^f^CBT: cognitive behavioral therapy.

^g^BAI: Beck Anxiety Inventory.

^h^iMBSR: internet-delivered mindfulness-based stress reduction.

^i^PHQ-15: Patient Health Questionnaire-15.

^j^BDI-II: Beck Depression Inventory–II.

^k^BA: behavioral activation.

^l^GAD-7: General Anxiety Disorder-7.

^m^PHQ-9: 9-item Patient Health Questionnaire.

^n^WHO: World Health Organization.

^o^ASHA: accredited social health activist.

^p^NABL: National Accreditation Board for Testing and Calibration Laboratories.

^q^PSS: Perceived Stress Scale.

^r^MITHRA: Multiuser Interactive Health Response App.

^s^HAP: healthy activity program.

^t^VR: virtual reality.

^u^ITT: intention to treat.

^v^SMD: standardized mean difference.

^w^CMD: common mental disorder.

^x^LMIC: low- and middle-income country.

^y^mHealth: mobile health.

^z^SVR: smartphone-based virtual reality.

^aa^AI: artificial intelligence.

^ab^PPD: postpartum depression.

^ac^EXPONATE: Expanding Care for Perinatal Women With Depression.

^ad^mhGAP-IG: Mental Health Gap Action Programme Intervention Guide.

^ae^PHC: primary health care.

^af^GPM+: group problem management plus.

^ag^HCW: health care worker.

^ah^mhGAP: Mental Health Gap Action Programme.

^ai^FIIT: fitness intervention.

^aj^ART: antiretroviral therapy.

^ak^RRMS: relapsing-remitting multiple sclerosis.

^al^MS: multiple sclerosis.

^am^SPMS: secondary progressive multiple sclerosis.

^an^AIIMS: All India Institute of Medical Science.

^ao^IMSD: Initiative Maternal Screening Depression.

^ap^DMHI: digital mental health intervention.

^aq^GIAI: guided internet-assisted intervention.

^ar^MD: mean difference.

^as^CHN: community health nurse.

^at^ASRaDA: Application for Suicide Risk and Depression Assessment.

^au^CES-D: Center for Epidemiologic Studies Depression Scale.

^av^SBQ-R: Suicide Behaviors Questionnaire–Revised.

^aw^BRT: Benson relaxation technique.

^ax^TA-PM+: technology-assisted problem management plus.

^ay^MAUQ: mHealth App Usability Questionnaire.

^az^SA: social anxiety.

^ba^PSA: public speaking anxiety.

^bb^PEERS: Promoting effective mental healthcare through peer supervision.

^bc^MDD: major depressive disorder.

^bd^CONEMO: Control and Emotional.

^be^iCBT: internet-delivered cognitive behavioral therapy.

^bf^AMISOM: African Union Mission in Somalia.

^bg^UN: United Nations.

### Synthesis of Results

#### Overview

This section presents a summary of the findings from the 80 articles that reported on fidelity, usability, and cultural adaptation of DMHIs, along with the challenges to and facilitators of their uptake in LMICs. The synthesis was organized by the three research objectives: (1) type of DMHI intervention platform; (2) discussions on their fidelity, usability, and cultural adaptation; and (3) identified challenges to and facilitators of their uptake in LMICs. A summary is presented in Table 2 organized according to the type of DMHI intervention platform used, along with other articles that reported the use of more than one type of DMHI.

**Table 2:** Summary of fidelity, usability, cultural adaptation, and challenges and facilitators by type of intervention.

	Fidelity	Usability	Cultural adaptation	Challenges	Facilitators
Summary (N=80)	A total of 16 articles reported on fidelity.	A total of 29 articles reported on usability.	A total of 31 articles reported on cultural adaptation.	A total of 22 articles reported on challenges to the uptake of DMHIs^a^ in LMICs^b^.	A total of 17 articles reported on facilitators of the uptake of DMHIs in LMICs.
Mobile apps (n=46)	A total of 12 articles reported on fidelity [57,64,70,79,80,83, 89,95,111,112,119,127].	A total of 23 articles reported on usability [53,57,62,64,65,67, 70,80,86,89, 90,93, 95,100,104,105,110-112, 115,117,119,120].	A total of 24 articles reported on cultural adaptation [48,53,64,67,68, 70,80,87,89,90,92, 93,95,104,105,108,110, 112,117,119,122,124,125].	A total of 13 articles reported on challenges [48,57,62,64,79, 80, 89,90,107,111,117,125, 127].	A total of 12 articles reported on facilitators [53,57,64,67,79, 83, 89,90,105,111,117,125].
Web-based programs (n=10)	One article reported on fidelity [55].	Two articles reported on usability [55,84].	Three articles reported on cultural adaptation [55,58,84].	Three articles reported on challenges [55,58,84].	Two articles reported on facilitators [55,58].
Virtual reality (n=5)	—^c^	—	One article reported on cultural adaptation [88].	—	—
Videoconferencing (n=4)	—	—	—	Two articles reported on challenges [66,98].	—
Telemedicine (n=4)	—	—	—	—	—
Social media–based interventions (n=1)	—	—	—	—	—
More than one delivery platform type (eg, delivered via app or the web or reviews that included various DMHIs; n=10)	Three articles reported on fidelity [71-73].	Four articles reported on usability [51,73,78,101].	Three articles reported on cultural adaptation [71,72,101].	Four articles reported on challenges [72,73,78,101].	Three articles reported on facilitators [49,73,101].

^a^DMHI: digital mental health intervention.

^b^LMIC: low- and middle-income country.

^c^Not applicable.

#### Types of Digital Health Interventions for Depression and Anxiety Being Used in LMICs

This search and review of the 80 articles showed that, of the DMHIs identified, the largest category was mobile apps (n=46, 58%). Other DMHIs involved web-based programs (n=10, 12%), VR (n=5, 6%), videoconferencing (n=4, 5%), telemedicine (interventions delivered via an instant messaging app; n=4, 5%), and social media–based interventions (n=1, 1%). A total of 12% (10/80) of the articles reported on delivery of mental health care using more than one platform (eg, delivered via app or the web or reviews that included various DMHIs). DMHIs can be stand-alone self-guided programs or incorporate a hybrid model whereby human assistance, either from a therapist (ie, mental health specialist) or a lay health worker to augment their care, is available with varying levels of support.

Most of the articles (64/80, 80%) reported strengths of DMHIs either on improving symptoms of depression or anxiety or other factors such as high level of usability, acceptability, and satisfaction during usability and pilot-testing. Overall, DMHIs show promise as a way to bridge the mental health treatment gap for depression and anxiety in LMICs.

#### Fidelity, Usability, and Cultural Adaptation

##### Fidelity

Despite the accelerated increase in the development and application of DMHIs in recent years, with many being adapted from existing in-person interventions, only 20% (16/80) of the articles reported on fidelity. Of these 16 articles, only 4 (25%) discussed fidelity in relation to the original in-person or paper-based intervention format. One of these articles mentioned adherence to the original intervention structure and format in the adapted digital format [80]. Another article described the process of involving psychologists and psychiatrists to ensure theoretical alignment of app content with psychosocial components [83]. The third article mentioned the development of 1 video per session to deliver the therapeutic ingredients with fidelity and at the right dose [57]. The last of the 4 articles mentioned that the intervention delivery format (mobile app) ensured that key therapeutic elements were delivered with fidelity and at the appropriate dose using the app’s automated features [64].

Most of the articles that discussed fidelity (13/80, 16%) described the process for ensuring fidelity or adherence by lay health workers to the intervention protocol. This included fidelity and audit checks of lay health workers by supervisors and reviews of case notes [55,70-73,79] and use of various tools (eg, fidelity checklist [71,72,79] or the practical robust implementation and sustainability model framework [95]). Regarding those reporting on the use of a tool, 2% (2/80) of the articles provided further information, indicating that 5% of the guidance calls and messages were rated (no additional information was provided on who performed the rating) [71,72]. Another article reported that the structured steps on the app made it easier for lay workers to adhere to fidelity when navigating the sessions [73], whereas another article indicated that the intervention itself, which was used for detection and treatment, could increase consistency in diagnosis and treatment [89]. Finally, one article stated that fidelity to the intervention protocol was demonstrated by lay health workers delivering the intervention adapting easily to the technology (smartphones) due to familiarity with the protocol [57]. Regarding the articles that reported on measuring fidelity of lay health workers to the intervention protocol, overall, it was shown that lay counselors adhered to the intervention delivery components.

##### Usability

Less than half (29/80, 36%) of the articles described usability in some manner, including through usability testing or qualitative exploration. Most of the articles (23/29, 79%) were related to mobile apps. Of the 29 articles, only 7 (24%) reported the use of measures, either standardized (eg, the System Usability Scale or mHealth App Usability Questionnaire [MAUQ]) or created by the authors for the study (eg, satisfaction survey), to capture usability [73,78,84,95,111,115]. In addition, only 31% (9/29) of the articles described the usability testing process along with findings in detail, with most (7/9, 78%) related to mobile apps [62,64,65,80,95,105,110,111,115] and the rest (2/9, 22%) pertaining to web-based programs [55,84]. In total, 10% (3/29) of the articles were protocol papers that indicated how app development would follow a user-centered iterative design.

Most of the articles that described usability testing outcomes (19/80, 24%) reported high satisfaction. For example, the study by Nurbaeti et al [100] showed that the evaluation of an app for postpartum depression symptoms in Indonesia was mostly positive, with a large percentage of the participants reporting the following: appearance of the app being adequate (92.7%) and the app being easy to download (89.9%), understandable (96.3%), easy to fill in (94.5%), and beneficial (96.3%) and reflecting psychological conditions (90.8%). Another article reporting the protocol for a feasibility cluster randomized controlled trial to be conducted in Nepal and Nigeria indicated that health workers found the prototypes of the app for the adapted WHO electronic Mental Health Gap Action Programme Intervention Guide to be intuitive, appropriate, and feasible for clinical work [112].

Of the 10 articles that reported more than one delivery platform, 4 (40%) reported on usability testing [51,73,78,101]. In total, 75% (3/4) of these articles indicated the evaluation of a usability-related construct using particular measures—a usability questionnaire (MAUQ) [73], satisfaction survey [78], and completion rates with participant feedback [51]. The remaining article mentioned that piloting had been conducted to evaluate the appropriateness of the intervention without describing this in detail [101]. Usability was reported as being high (eg, the MAUQ score was 78.5, and the satisfaction survey showed that 92% of participants were very satisfied).

##### Cultural Adaptation

Finally, 39% (31/80) of the articles described a process for adapting the DMHIs to the local cultural context. Of these 31 articles, 23 (74%) reported on mobile apps. Various methods were used, including a narrative approach involving local women, a participatory approach, identification of measures that had been validated in the implementation country or working with local partners to enhance cultural validity where needed, and the intervention being led by a local partner. Many of the articles (13/80, 16%) discussed the involvement of a broad range of stakeholders in the adaptation process using a “participatory approach” [67]. For example, one article mentioned how “the vignettes were developed in consultation with local women who had suffered from depression” [64]. Another article described that, to improve cultural fit, “a team of clinical psychologists, psychiatrists, and researchers from the U.S. and India adapted the intervention,” and “modifications were informed by student feedback” [84].

A few articles (7/80, 9%) described the cultural adaptation process in detail. For example, one article described how the VR space was designed to be similar to the environment of the end users (urban refugee youths in Kampala) and presented in their language of choice combined with training materials and content adapted with peer navigators to enhance relevance [88]. Another article detailed the extensive cultural adaption process conducted by the WHO and the Lebanon National Mental Health Programme to develop relevant content (story, local idioms, and illustrations) for the Lebanese population, displaced Syrians, and other groups in Lebanon. The article also included descriptions of piloting for face and content validity, clarifying language, assessing readability, and establishing content appropriateness within the intended context [55].

Most of the articles (18/80, 20%) were vaguer, indicating simply the importance of identifying what would be culturally acceptable, that the app was designed for the specific population, or the use of a guide adapted for the health system of the target country. A total of 2% (2/80) of the articles, which reported on web-based DMHIs, indicated that cultural adaptation was conducted with the intervention or culturally adapted approaches were used, resulting in high cultural relevance and acceptability; however, the process was not described in detail [58,84]. Similarly, 67% (2/3) of the articles that reported more than one delivery platform mentioned cultural adaptation but did not describe the process in detail [71,72]. These 2 articles pertained to the same intervention. Another article mentioned that the narrative was adapted to the local context considering cultural and linguistic nuances among displaced Syrians and that participants could choose the appearance of the avatar used in the program, reflecting the main cultural groups in Lebanon (Lebanese, Syrians, and Palestinians) [64].

#### Challenges to and Facilitators of Intervention Uptake in LMICs

##### Challenges to Uptake

A total of 28% (22/80) of the articles reported on challenges. In total, 59% (13/22) of these articles were related to mobile apps, describing challenges that pertained to the technological system (eg, system lags, app navigation challenges, and bugs); user engagement issues (sustained engagement of individuals who are depressed and may have low motivation, high churn, and issues with adherence to treatment), which are linked to poor usability; contextual issues (eg, illiteracy, technological proficiency, accessibility of mobile phones, data limits, battery charging issues, and lack of family involvement); structural barriers (eg, network connectivity issues and resource constraints); and concerns about privacy and confidentiality [48,57,62,64,79,80,89,90,107,111,117,125,127]. Of the 22 articles, 2 (9%) highlighted how many of these challenges contribute to the digital divide, or the division between individuals or populations who have access to and use digital technology and those who do not [64,117]. Other challenges mentioned included that of a practice gap between health care providers and application developers [48] and challenges with the online delivery method where participants complete tasks in their own time [79].

In addition to similar challenges described previously, other challenges reported in relation to web-based programs and those delivered via more than one platform included individuals forgetting their log-in credentials, time constraints due to busy schedule of the participants, retention of lay health workers, sustained engagement, and stigma [55,66,84]. There were also concerns reported about the interventions’ efficacy, effectiveness, and usability [73].

##### Facilitators of Uptake

In total, 21% (17/80) of the articles reported on facilitators. Of these 17 articles, 12 (71%) were related to mobile apps, describing various facilitators that supported their uptake and scale-up, including widespread mobile phone use, built-in supervision and training features, and an app’s convenience and integration into existing social (eg, schools) and health care systems [53,57,64,67,79,83,89,90,105,111,117,125]. One of these studies suggested a web-based delivery platform rather than an app-based one as the former does not require regular updating and tailored interventions or distinct adaptations that account for the unique needs of the target population [79]. Other articles (3/80, 4%) reported on the importance of orientation and training programs and mobilization of community health volunteers [89,111] and how an understanding of the intervention and its potential benefits will likely support its uptake [90]. In line with some of the concerns that clinicians expressed, described previously, one article reported on the importance of buy-in from frontline health care providers and clinical experts, which may be facilitated by demonstrated clinical utility and efficiency [117]. Other facilitators reported in relation to web-based programs or interventions involving more than one delivery platform included facilitators at the individual level, such as being technologically savvy and literate and having access to devices [55], and incorporating features to boost user engagement [101]. Finally, the importance of cultural appropriateness was emphasized by a number of the articles (11/80, 14%; see, eg, the studies by Alavi and Hirji [58] and Pozuelo et al [105]).

## Discussion

### Principal Findings

The 80 articles identified in this rapid scoping review reported the use of mobile apps, web-based programs, VR, videoconferencing, telemedicine (interventions delivered via instant messaging apps) platforms, and social media, in addition to instances in which the DMHIs were delivered via more than one platform (eg, delivered via app or the web). To our knowledge, this rapid scoping review is the most comprehensive presentation of articles examining DMHIs for depression and anxiety and their fidelity, usability, and cultural adaptation, along with challenges to and facilitators of their uptake in LMICs.

#### Fidelity

Active elements of these DMHIs are often modeled on evidence-based psychological interventions developed for in-person delivery [17], offering further promise to provide scalable effective mental health care. As such, it is critical that the interventions are implemented with high fidelity while balancing the need for necessary adaptations to the digital context, including the different motivational needs and preferences of digital users. However, inconsistencies regarding treatment fidelity have been identified as a main contributor to the mixed evidence on the effectiveness of many DMHIs [128]. Similarly, results from this review showed that less than a fifth (16/80, 20%) of the articles reported on fidelity, with heterogeneity in process and presentation format. Most of these articles (13/80, 16%) described the process for ensuring adherence by lay health workers to the intervention protocol rather than for ensuring fidelity of the DMHI to its original in-person or paper-based format. Furthermore, only 1% (1/80) of the articles mentioned specifically adherence of the DMHI to the original intervention structure albeit delivered in an automated digital format [80]. Interestingly, 2% (2/80) of the articles mentioned that the delivery format of the intervention (app) supported fidelity via the nature of its automated and structured format [64,89]. This is consistent with previous research demonstrating that less complex interventions are easier to implement with high fidelity [129]. As more DHMIs are being developed and adapted from in-person or paper-based formats, it is crucial to ensure intervention fidelity to maintain consistency in intervention delivery across platforms while balancing necessary adaptations to suit digital modes of delivery and the local context [130,131].

#### Usability

Poor usability has been cited as a primary reason for failed adoption of and engagement with digital health apps [31,32], where approximately one-third of users who download health apps stop using them after a short time, often no more than 2 weeks [132]. Hurdles to app usability include being difficult to set up or use or being buggy, not working well, requiring too much time to enter data, or having small screens that display limited information at a time [32,133]. Other challenges include concerns about privacy. An additional consideration for individuals with depression include specific symptoms such as low motivation, concentration difficulties, and behavioral avoidance that may make it more difficult for them to engage with DMHIs, posing further challenges to real-world efficacy [133]. Although usability testing is understood increasingly as an important component of app development and evaluation [134], usability currently remains underexplored in depression app development [135,136]. Findings from this review reflect this as only 36% (29/80) of the articles described usability testing in some manner despite overwhelming evidence highlighting how the efficacy of mobile apps can be greatly enhanced by conducting usability testing to determine factors impacting user adherence and satisfaction [137-139].

Articles that reported on usability outcomes (9/80, 11%) demonstrated mostly positive findings, with above-average usability and satisfaction scores being reported. Certain challenges to usability were also reported, including a confusing registration process and content that could be improved to be more engaging [80], which was a barrier to adherence [55]. Despite the generally positive usability and satisfaction scores reported by the various articles, poor usability has been clearly identified as a barrier to sustained user engagement across various digital health interventions [140,141]. A lack of user engagement with the DMHI regardless of users’ self-reports on satisfaction and utility likely indicates poor usability [142] as usability is a precursor for engagement [143]. This is also reflected in the fact that, despite the large number of medical health apps available, their overall downloads are decreasing [31]. Methods to increase usability include incorporating interactive features (eg, tooltips) [144] and gamification (eg, badges and avatars) [145]. Usability is also intricately linked with acceptability, which includes the intuitive attitudes toward the intervention, use intentions, actual use, and satisfaction after using [146]. Acceptability is intricately linked with prevailing social and cultural norms [147], highlighting further the importance of cultural adaptation.

#### Cultural Adaptation

Ensuring that interventions are culturally appropriate and relevant is important to their success [148,149]. While depression and anxiety affect people from all cultures, the experience of these illnesses is culturally bound, shaped by local beliefs, practices, and norms. This cultural variation profoundly influences mental illness expression; patterns of coping and help seeking; the process of recovery; adherence to treatment; and relationships between individuals and their networks of family, friends, and health care providers [150,151]. Cultural adaptations to increase relevance for individuals from ethnic minority groups have been shown to have greater efficacy [152] and more favorable health outcomes [153].

However, there has been limited focus on cultural adaptations of DMHIs to ensure relevance and acceptability [19,20]. Most of the 39% (31/80) of the articles that reported on cultural adaptation only touched on the cultural adaptation process undertaken, for example, “clarify[ing] language, and establish[ing] the appropriateness of the developed content within the intended context of use” [101] and “the narrative was adapted to the local context, considering gender, linguistic, and cultural nuances” [71,72]. Only 9% (7/80) of the articles described the cultural adaptation process in detail. While it is encouraging that many of the articles included in this review (31/80, 39%) discussed cultural adaptation in some form, there is a need for researchers and developers to place more emphasis on the cultural adaptation process to ensure that evidence-based interventions are properly adapted to enhance favorable health outcomes.

Promisingly, a number of the articles (13/80, 16%) discussed the involvement of a broad range of stakeholders in the development and cultural adaptation process. Engaging a broad range of stakeholders, including experts, end users, and community members, has been shown to contribute critical insights for development, implementation, and evaluation [153]. Developing DMHIs that address the culture and context of the diverse groups in LMICs by including necessary cultural adaptations while maintaining intervention fidelity is critical to increasing engagement of historically underserved cultural groups [21].

#### Challenges and Facilitators

A total of 28% (22/80) of the articles reported on challenges, whereas 21% (17/80) reported on facilitators. Unlike the topics of fidelity, usability, and cultural adaptation, where most of the articles discussed mobile apps, articles that discussed challenges and facilitators were spread more evenly across the different delivery platforms, highlighting the consistency of challenges and facilitators experienced with different types of DMHIs. Many of the challenges reported in the articles (eg, contextual issues and structural barriers) were perceived as possibly leading to inequitable digital access and contributing further to the digital divide. These findings are consistent with the literature [12], which indicates that, while DMHIs offer an accessible and promising method for delivering mental health care, a number of challenges remain from the end user and developer side that must be addressed. For example, many of the individuals who use these types of DMHIs may live in rural areas where contextual (eg, being busy with multiple responsibilities such as farming, taking care of family, and side jobs) and structural issues (underdeveloped technological infrastructure) may prevent them from engaging fully with DMHIs. Structural issues and digital health literacy have been widely reported in the literature for a number of years [154,155]. A lack of digital literacy, especially among the older adult population and those from lower socioeconomic brackets, is one of the main challenges in scaling up digital health interventions [155,156]. In addition, DMHIs may not be accessible for subpopulations such as racial and ethnic minority groups due to language barriers [157], emphasizing again the importance of ensuring cultural appropriateness. Finally, for DMHIs that incorporate a hybrid model involving human assistance, in particular lay health workers to augment their care, the articles emphasized the heavy burden on the providers, who often have heavy existing workloads and are largely underpaid or even unpaid [158]. This may pose a challenge for sustainability.

Clinician buy-in was reported as a challenge to the overall implementation of DMHIs into routine care and centered on time management, regulatory structures and licensure, lack of nonverbal evidence, and payment. Similar challenges have been reported in the literature, demonstrating that there has been a reticence from mental health professionals to engage with DMHIs due to the perceived threat to the therapeutic alliance, which some believe can only be established in person [25,26]. Research suggests that there is presently limited theoretical and practical training on novel digital tools and interventions in psychiatry, which influences clinicians’ understanding and perceptions of DMHIs [159]. The importance of in-person contact in psychotherapy has also been widely discussed in the literature [160-162]. However, Newman et al [163] showed that, while clinician-assisted treatments are indeed optimal, interventions with reduced in-person contact and at reduced intensity have also been shown to be effective and cost-effective. This has been researched most heavily in the case of mobile apps [164,165]. For example, 2 systematic reviews of mobile apps (both self-guided and hybrid models) demonstrated their effectiveness in reducing depression symptoms [16,139]. Nevertheless, clinician concerns need to be addressed to enhance their acceptance of and engagement with DMHIs, particularly for those that are integrated into standard care and may require their support. One method may be engaging clinicians more throughout the app development process to provide expert input and also increase their understanding and acceptance of DMHIs [13].

Facilitators of the uptake and scale-up of DMHIs in LMICs included widespread mobile phone use, built-in supervision and training features, their convenience, and integration into existing social and health care systems. For the hybrid models that required lay health workers for ongoing implementation, mobilization of community health volunteers, including through training and professional support, was considered an important facilitator. Training and professional support have been shown to increase lay health workers’ satisfaction and enhance their agency and social status [166]. Ensuring cultural appropriateness to the local context by using tailored adaptations was also emphasized. This can increase the reach of and engagement with DMHIs and increase equity by avoiding the systematic exclusion of cultural groups who have been historically underserved [21]. Furthermore, increased mental health awareness was highlighted as an important facilitator to increase understanding of the DMHI and its potential benefits. In addition to demonstrated clinical effectiveness of DMHIs for depression and anxiety, their use has been shown to increase mental health literacy and awareness [154]. Finally, the importance of buy-in from clinicians and other providers was highlighted. In addition to DMHI integration into standard care, clinician behaviors could support adherence. For example, Paxling et al [167] demonstrated that certain clinician behaviors, such as task reinforcement, task prompting, self-efficacy shaping, and empathetic utterances, were correlated with module completion [168].

### Limitations

A limitation of this review is that we only included English-language papers, leading to an unknown number of articles from LMICs published in languages other than English being excluded. However, we believe that this review of 80 articles identified many of the key issues concerning DMHIs in LMICs. A second limitation is that this review focused only on depression and anxiety; examination of DMHIs for other mental health conditions may elucidate additional considerations. However, as depression and anxiety are the 2 most common mental health conditions [5] and many DMHIs are developed for depression and anxiety [6,15,169], the results of this review can inform the further development and delivery of DMHIs for these 2 conditions and provide information on general rather than condition-specific features of DMHIs focusing on other conditions. Finally, research published before the search time frame may have contributed additional findings. Considering the rapid advancement of DMHIs and related research stimulated by the COVID-19 pandemic, we believe that this review of research published during the applied time frame captured the most crucial and timely evidence.

Given the rapidly changing landscape of development and application of DMHIs, further research is warranted to capture emergent changes, including the interventions’ fidelity, usability, and cultural appropriateness and challenges to and facilitators of their uptake in LMICs. Additional future research should also focus on underexplored areas, including an examination of the percentage of DMHIs that have no basis in evidence; the potential for harms in the unrestrained development and delivery of DMHIs; and the threats to privacy and security, such as potential for financial harm through unregulated mobile apps, including those presented as DMHIs. Finally, the accelerated growth of artificial intelligence (AI) and its increasing application in various health care fields in recent years could transform the landscape of digital mental health, including DMHIs [168,170]. While AI represents a potential means to advance mental health care, there will be an ensuing urgent need to examine in detail ethical implications associated with the integration of AI into digital mental health [171].

### Conclusions

DMHIs are one of the most promising ways to better meet population mental health needs and respond in culturally appropriate ways to diverse population groups. Despite the opportunities they offer for reducing the severe and persistent mental health treatment gap, there were several key issues identified in this scoping review that warrant further examination. These include further work on the fidelity, user engagement, and cultural adaptation of DMHIs. In addition, various challenges to the uptake of DMHIs in LMICs, including challenges pertaining to the technological system, contextual issues, structural barriers, and concerns about privacy and confidentiality, must be mitigated in the ongoing implementation of DMHIs in LMICs to avoid contributing further to the digital divide. In summary, DMHIs in isolation are not a solution to address the significant mental health care system deficiencies that are present; rather, they may serve as a low-cost and low-barrier treatment option—especially those DMHIs that are appropriately adapted from evidence-based in-person interventions—in low-resource settings where community-based care is severely limited.

## Appendix

Multimedia Appendix 1 Final PubMed search strategy.

Multimedia Appendix 2 Final PsycINFO search strategy.

Multimedia Appendix 3 PRISMA-ScR (Preferred Reporting Items for Systematic Reviews and Meta-Analyses extension for Scoping Reviews) checklist.

Multimedia Appendix 4 Summary of the excluded articles after full-text review.

## Abbreviations

AI: artificial intelligence
DMHI: digital mental health intervention
LMIC: low- and middle-income country
MAUQ: mHealth App Usability Questionnaire
PRISMA: Preferred Reporting Items for Systematic Reviews and Meta-Analyses
PRISMA-ScR: Preferred Reporting Items for Systematic Reviews and Meta-Analyses extension for Scoping Reviews
VR: virtual reality
WHO: World Health Organization

## References

[1] GBD 2019 Mental Disorders Collaborators (2022). Global, regional, and national burden of 12 mental disorders in 204 countries and territories, 1990-2019: a systematic analysis for the Global Burden of Disease Study 2019. Lancet Psychiatry.

[2] Torous J, Jän Myrick K, Rauseo-Ricupero N, Firth J (2020). Digital mental health and COVID-19: using technology today to accelerate the curve on access and quality tomorrow. JMIR Ment Health.

[3] Zheng J, Morstead T, Sin N, Klaiber P, Umberson D, Kamble S, DeLongis A (2021). Psychological distress in North America during COVID-19: the role of pandemic-related stressors. Soc Sci Med.

[4] COVID-19 Mental Disorders Collaborators (2021). Global prevalence and burden of depressive and anxiety disorders in 204 countries and territories in 2020 due to the COVID-19 pandemic. Lancet.

[5] Patel V, Saxena S, Lund C, Thornicroft G, Baingana F, Bolton P, Chisholm D, Collins PY, Cooper JL, Eaton J, Herrman H, Herzallah MM, Huang Y, Jordans MJ, Kleinman A, Medina-Mora ME, Morgan E, Niaz U, Omigbodun O, Prince M, Rahman A, Saraceno B, Sarkar BK, De Silva M, Singh I, Stein DJ, Sunkel C, UnÜtzer J (2018). The Lancet Commission on global mental health and sustainable development. Lancet.

[6] Linardon J, Torous J, Firth J, Cuijpers P, Messer M, Fuller-Tyszkiewicz M (2024). Current evidence on the efficacy of mental health smartphone apps for symptoms of depression and anxiety. A meta-analysis of 176 randomized controlled trials. World Psychiatry.

[7] World Bank country and lending groups. The World Bank.

[8] Ahmed O, Ahmed MZ, Alim SM, Khan MD, Jobe MC (2022). COVID-19 outbreak in Bangladesh and associated psychological problems: an online survey. Death Stud.

[9] Mendes-Santos C, Andersson G, Weiderpass E, Santana R (2020). Mitigating COVID-19 impact on the Portuguese population mental health: the opportunity that lies in digital mental health. Front Public Health.

[10] Mental Health Gap Action Programme (mhGAP). World Health Organization.

[11] Carter H, Araya R, Anjur K, Deng D, Naslund JA (2021). The emergence of digital mental health in low-income and middle-income countries: A review of recent advances and implications for the treatment and prevention of mental disorders. J Psychiatr Res.

[12] Chakrabarti S (2024). Digital psychiatry in low-and-middle-income countries: new developments and the way forward. World J Psychiatry.

[13] Philippe TJ, Sikder N, Jackson A, Koblanski ME, Liow E, Pilarinos A, Vasarhelyi K (2022). Digital health interventions for delivery of mental health care: systematic and comprehensive meta-review. JMIR Ment Health.

[14] Torous J, Linardon J, Goldberg SB, Sun S, Bell I, Nicholas J, Hassan L, Hua Y, Milton A, Firth J (2025). The evolving field of digital mental health: current evidence and implementation issues for smartphone apps, generative artificial intelligence, and virtual reality. World Psychiatry.

[15] Firth J, Torous J, Nicholas J, Carney R, Pratap A, Rosenbaum S, Sarris J (2017). The efficacy of smartphone-based mental health interventions for depressive symptoms: a meta-analysis of randomized controlled trials. World Psychiatry.

[16] Massoudi B, Holvast F, Bockting CL, Burger H, Blanker MH (2019). The effectiveness and cost-effectiveness of e-health interventions for depression and anxiety in primary care: a systematic review and meta-analysis. J Affect Disord.

[17] Murray E, Hekler EB, Andersson G, Collins LM, Doherty A, Hollis C, Rivera DE, West R, Wyatt JC (2016). Evaluating digital health interventions: key questions and approaches. Am J Prev Med.

[18] Dunst CJ, Trivette CM, Raab M (2013). An implementation science framework for conceptualizing and operationalizing fidelity in early childhood intervention studies. J Early Interv.

[19] Eustis EH, LoPresti J, Aguilera A, Schueller SM (2023). Cultural responsivity in technology-enabled services: integrating culture into technology and service components. J Med Internet Res.

[20] Lal S, Gleeson J, Malla A, Rivard L, Joober R, Chandrasena R, Alvarez-Jimenez M (2018). Cultural and contextual adaptation of an eHealth intervention for youth receiving services for first-episode psychosis: adaptation framework and protocol for horyzons-Canada phase 1. JMIR Res Protoc.

[21] Nittas V, Daniore P, Chavez SJ, Wray TB (2024). Challenges in implementing cultural adaptations of digital health interventions. Commun Med (Lond).

[22] Naslund JA, Spagnolo J, Jacobson N, Kowatsch T, Marsch L (2022). Cultural adaptations of digital therapeutics. Digital Therapeutics for Mental Health and Addiction: The State of the Science and Vision for the Future.

[23] Bernal G, Jiménez-Chafey MI, Domenech Rodríguez MM (2009). Cultural adaptation of treatments: a resource for considering culture in evidence-based practice. Prof Psychol Res Pr.

[24] Wind TR, Rijkeboer M, Andersson G, Riper H (2020). The COVID-19 pandemic: the 'black swan' for mental health care and a turning point for e-health. Internet Interv.

[25] Topooco N, Riper H, Araya R, Berking M, Brunn M, Chevreul K, Cieslak R, Ebert DD, Etchmendy E, Herrero R, Kleiboer A, Krieger T, García-Palacios A, Cerga-Pashoja A, Smoktunowicz E, Urech A, Vis C, Andersson G, E-COMPARED consortium (2017). Attitudes towards digital treatment for depression: a European stakeholder survey. Internet Interv.

[26] Marshall JM, Dunstan DA, Bartik W (2020). Clinical or gimmickal: the use and effectiveness of mobile mental health apps for treating anxiety and depression. Aust N Z J Psychiatry.

[27] Al Dweik R, Ajaj R, Kotb R, Halabi DE, Sadier NS, Sarsour H, Elhadi YA (2024). Opportunities and challenges in leveraging digital technology for mental health system strengthening: a systematic review to inform interventions in the United Arab Emirates. BMC Public Health.

[28] Torous J, Myrick K, Aguilera A (2023). The need for a new generation of digital mental health tools to support more accessible, effective and equitable care. World Psychiatry.

[29] Hasegawa K, O'Brien N, Prendergast M, Ajah CA, Neves AL, Ghafur S (2024). Cybersecurity interventions in health care organizations in low- and middle-income countries: scoping review. J Med Internet Res.

[30] The V3+ usability validation glossary. Datacc.

[31] Liew MS, Zhang J, See J, Ong YL (2019). Usability challenges for health and wellness mobile apps: mixed-methods study among mHealth experts and consumers. JMIR Mhealth Uhealth.

[32] Torous J, Nicholas J, Larsen ME, Firth J, Christensen H (2018). Clinical review of user engagement with mental health smartphone apps: evidence, theory and improvements. Evid Based Ment Health.

[33] Lipschitz JM, Van Boxtel R, Torous J, Firth J, Lebovitz JG, Burdick KE, Hogan TP (2022). Digital mental health interventions for depression: scoping review of user engagement. J Med Internet Res.

[34] Breuer E, Hanlon C, Bhana A, Chisholm D, Silva MD, Fekadu A, Honikman S, Jordans M, Kathree T, Kigozi F, Luitel NP, Marx M, Medhin G, Murhar V, Ndyanabangi S, Patel V, Petersen I, Prince M, Raja S, Rathod SD, Shidhaye R, Ssebunnya J, Thornicroft G, Tomlinson M, Wolde-Giorgis T, Lund C (2019). Partnerships in a global mental health research programme-the example of PRIME. Glob Soc Welf.

[35] Chibanda D, Abas M, Musesengwa R, Merritt C, Sorsdahl K, Mangezi W, Bandawe C, Cowan F, Araya R, Gomo E, Gibson L, Weiss H, Hanlon C, Lund C (2020). Mental health research capacity building in sub-Saharan Africa: the African Mental Health Research Initiative. Glob Ment Health (Camb).

[36] Data for lower middle-income, low-income countries. The World Bank.

[37] Kola L, Kohrt BA, Hanlon C, Naslund JA, Sikander S, Balaji M, Benjet C, Cheung EY, Eaton J, Gonsalves P, Hailemariam M, Luitel NP, Machado DB, Misganaw E, Omigbodun O, Roberts T, Salisbury TT, Shidhaye R, Sunkel C, Ugo V, van Rensburg AJ, Gureje O, Pathare S, Saxena S, Thornicroft G, Patel V (2021). COVID-19 mental health impact and responses in low-income and middle-income countries: reimagining global mental health. Lancet Psychiatry.

[38] Li J (2023). Digital technologies for mental health improvements in the COVID-19 pandemic: a scoping review. BMC Public Health.

[39] Zhang M, Smith HE (2020). Digital tools to ameliorate psychological symptoms associated with COVID-19: scoping review. J Med Internet Res.

[40] Zhong S, Yang X, Pan Z, Fan Y, Chen Y, Yu X, Zhou L (2023). The usability, feasibility, acceptability, and efficacy of digital mental health services in the COVID-19 pandemic: scoping review, systematic review, and meta-analysis. JMIR Public Health Surveill.

[41] Tricco AC, Antony J, Zarin W, Strifler L, Ghassemi M, Ivory J, Perrier L, Hutton B, Moher D, Straus SE (2015). A scoping review of rapid review methods. BMC Med.

[42] Peters MD, Godfrey CM, Khalil H, McInerney P, Parker D, Soares CB (2015). Guidance for conducting systematic scoping reviews. Int J Evid Based Healthc.

[43] Brien SE, Lorenzetti DL, Lewis S, Kennedy J, Ghali WA (2010). Overview of a formal scoping review on health system report cards. Implement Sci.

[44] Arksey H, O'Malley L (2005). Scoping studies: towards a methodological framework. Int J Soc Res Methodol.

[45] Tricco AC, Langlois EV, Straus SE (2017). Rapid reviews to strengthen health policy and systems: a practical guide. World Health Organization.

[46] Moher D, Liberati A, Tetzlaff J, Altman DG, PRISMA Group (2009). Preferred reporting items for systematic reviews and meta-analyses: the PRISMA statement. PLoS Med.

[47] Campbell SM (2023). A filter to identify countries with low or lower middle income economies in the MEDLINE database. Geoffrey & Robyn Sperber Health Sciences Library, University of Alberta.

[48] Dosani A, Arora H, Mazmudar S (2020). mHealth and perinatal depression in low-and middle-income countries: a scoping review of the literature. Int J Environ Res Public Health.

[49] Fu Z, Burger H, Arjadi R, Bockting CL (2020). Effectiveness of digital psychological interventions for mental health problems in low-income and middle-income countries: a systematic review and meta-analysis. Lancet Psychiatry.

[50] Kim J, Aryee LM, Bang H, Prajogo S, Choi YK, Hoch JS, Prado EL (2023). Effectiveness of digital mental health tools to reduce depressive and anxiety symptoms in low- and middle-income countries: systematic review and meta-analysis. JMIR Ment Health.

[51] van der Watt AS, Odendaal W, Louw K, Seedat S (2020). Distant mood monitoring for depressive and bipolar disorders: a systematic review. BMC Psychiatry.

[52] Sarpourian F, Samad-Soltani T, Moulaei K, Bahaadinbeigy K (2022). The effect of virtual reality therapy and counseling on students' public speaking anxiety. Health Sci Rep.

[53] Aalaei S, Khoshrounejad F, Saleh LA, Amini M (2021). Design of a mobile application and evaluation of its effects on psychological parameters of COVID-19 inpatients: a protocol for a randomized controlled trial. Front Psychiatry.

[54] Abbasalizadeh M, Farsi Z, Sajadi SA, Atashi A, Fournier A (2024). The effect of resilience training with mHealth application based on micro-learning method on the stress and anxiety of nurses working in intensive care units: a randomized controlled trial. BMC Med Educ.

[55] Abi Ramia J, Abi Hana R, Noun P, Cuijpers P, Carswell K, Van't Hof E, Heim E, Zoghbi E, Sijbrandij M, El Chammay R (2023). Feasibility and uptake of a digital mental health intervention for depression among Lebanese and Syrian displaced people in Lebanon: a qualitative study. Front Public Health.

[56] Akbari A, Dalvand S, Ahmadi F, Khazaei S (2022). Impact of virtual training of Jacobson and Benson relaxation techniques on the anxiety among home-isolated COVID-19 patients. Tanaffos.

[57] Akhtar S, Rabbani F, Nafis J, Siddiqui A, Merali Z (2025). A qualitative study assessing acceptability and appropriateness of a technology-assisted mental health intervention by community frontline workers: mPareshan implementation research in rural Pakistan. BMC Psychiatry.

[58] Alavi N, Hirji A (2020). The efficacy of PowerPoint-based CBT delivered through email: breaking the barriers to treatment for generalized anxiety disorder. J Psychiatr Pract.

[59] Al-Badiri MQ, Ghadirian F, Zahednezhad H, Boozari M, Hayati M (2024). Comparison of the effects of in-person and internet-delivered mindfulness-based stress reduction on the burden of psychosomatic symptoms in nurses. Front Psychol.

[60] Alishahi M, Mohajer S, Namazinia M, Mazloum S (2025). The effect of recreational therapy application on depression in hemodialysis patients: a randomized clinical trial. Int Urol Nephrol.

[61] Amer NA, Shohieb SM, Eladrosy WM, Elbakry H, Elrazek S (2023). Sokoon: a gamification-based cognitive behavioral therapy application? An application for depression, stress, and anxiety. Int J Gaming Comput Mediat Simul.

[62] Amer NA, Shohieb SM, Eladrosy W, Liu S, Nam Y, Abdelrazek S (2024). A gamified cognitive behavioral therapy for Arabs to reduce symptoms of depression and anxiety: a case study research. Digit Health.

[63] Araya R, Menezes PR, Claro HG, Brandt LR, Daley KL, Quayle J, Diez-Canseco F, Peters TJ, Vera Cruz D, Toyama M, Aschar S, Hidalgo-Padilla L, Martins H, Cavero V, Rocha T, Scotton G, de Almeida Lopes IF, Begale M, Mohr DC, Miranda JJ (2021). Effect of a digital intervention on depressive symptoms in patients with comorbid hypertension or diabetes in Brazil and Peru: two randomized clinical trials. JAMA.

[64] Atif N, Nazir H, Sultan ZH, Rauf R, Waqas A, Malik A, Sikander S, Rahman A (2022). Technology-assisted peer therapy: a new way of delivering evidence-based psychological interventions. BMC Health Serv Res.

[65] Bhardwaj P, Joshi NK, Gupta MK, Sharma SK, Nebhinani N, Sinha A, Tyrrell M, Konradsen H, Craftman Å, Kabir ZN (2025). mHealth-based intervention by community workers to support family caregivers of persons with dementia living at home: study protocol for a cluster randomised controlled trial. BMJ Open.

[66] Bhargav H, Raghavan V, Rao NP, Gulati K, Binumon KV, Anu KN, Ravi S, Jasti N, Holla B, Varambally S, Ramachandran P (2024). Validation and efficacy of a tele-yoga intervention for improving psychological stress, mental health and sleep difficulties of stressed adults diagnosed with long COVID: a prospective, multi-center, open-label single-arm study. Front Psychol.

[67] Bhat A, Goud BR, Kalidindi B, Ruben JP, Devadass D, Waghmare A, Collins PY, Raj T, Srinivasan K (2023). Mobile mental health in women's community-based organizations: protocol for a pilot randomized controlled trial. JMIR Res Protoc.

[68] van Heerden A, Poudyal A, Kohrt BA, Maharjan SM, Hagaman A, Byanjankar P (2021). Utilizing passive sensing data to provide personalized psychological care in low-resource settings. Gates Open Res.

[69] Chand K, Chandra S, Dutt V (2024). Raga Bhairavi in virtual reality reduces stress-related psychophysiological markers. Sci Rep.

[70] Chau LW, Murphy JK, Nguyen VC, Xie H, Lam RW, Minas H, Zheng Y, Krebs E, Hayashi K, Dao S, Nguyen X, Duong VA, Fiume E, O'Neil J (2023). Evaluating the effectiveness and cost-effectiveness of a digital, app-based intervention for depression (VMood) in community-based settings in Vietnam: protocol for a stepped-wedge randomized controlled trial. PLoS One.

[71] Cuijpers P, Heim E, Ramia JA, Burchert S, Carswell K, Cornelisz I, Knaevelsrud C, Noun P, van Klaveren C, Van't Hof E, Zoghbi E, van Ommeren M, El Chammay R (2022). Guided digital health intervention for depression in Lebanon: randomised trial. Evid Based Ment Health.

[72] Cuijpers P, Heim E, Abi Ramia J, Burchert S, Carswell K, Cornelisz I, Knaevelsrud C, Noun P, van Klaveren C, Van't Hof E, Zoghbi E, van Ommeren M, El Chammay R (2022). Effects of a WHO-guided digital health intervention for depression in Syrian refugees in Lebanon: a randomized controlled trial. PLoS Med.

[73] Dambi J, Norman C, Doukani A, Potgieter S, Turner J, Musesengwa R, Verhey R, Chibanda D (2022). A digital mental health intervention (Inuka) for common mental health disorders in Zimbabwean adults in response to the COVID-19 pandemic: feasibility and acceptability pilot study. JMIR Ment Health.

[74] Doosti P, Etemadifar S, Aliakbari F (2024). The impact of a continuous care model utilizing a smartphone application on quality of life and anxiety levels among gynecologic cancer patients: a randomized controlled trial. BMC Nurs.

[75] Ebrahimi A, Akbarzadeh F, Asgharipour N, Salehabadi R, Arjamandi M (2025). Investigating the effectiveness of ailurophobia treatment using virtual reality technique compared to metacognitive therapy: a randomized clinical trial. BMC Psychol.

[76] Ganjekar S, Harve VS, Bhargav H, Kukreti P, Dere S, Thukral U, Thamke P, Puri M, Krishnamurthy MN (2024). The pregnancy tele-yoga module to combat stress, anxiety, and depression associated with pregnancy: an exploratory open-label multicentric study. Int J Yoga.

[77] Gautama MS, Haryani H, Huang T, Chen J, Chuang Y (2024). Effectiveness of smartphone-based virtual reality relaxation (SVR) for enhancing comfort in cancer patients undergoing chemotherapy: a randomized controlled trial. Support Care Cancer.

[78] Ghanbari E, Yektatalab S, Mehrabi M (2021). Effects of psychoeducational interventions using mobile apps and mobile-based online group discussions on anxiety and self-esteem in women with breast cancer: randomized controlled trial. JMIR Mhealth Uhealth.

[79] Gonsalves PP, Bhat B, Sharma R, Jambhale A, Chodankar B, Verma M, Hodgson E, Weiss HA, Leurent B, Cavanagh K, Fairburn CG, Cuijpers P, Michelson D, Patel V (2022). Pilot randomised controlled trial of a remotely delivered online intervention for adolescent mental health problems in India: lessons learned about low acceptability and feasibility during the COVID-19 pandemic. BJPsych Open.

[80] Green EP, Lai Y, Pearson N, Rajasekharan S, Rauws M, Joerin A, Kwobah E, Musyimi C, Jones RM, Bhat C, Mulinge A, Puffer ES (2020). Expanding access to perinatal depression treatment in Kenya through automated psychological support: development and usability study. JMIR Form Res.

[81] Gupta S, Goel L, Singh A, Prasad A, Ullah MA (2022). Psychological analysis for depression detection from social networking sites. Comput Intell Neurosci.

[82] Iravani M, Bahmaei H, Askari S, Ghanbari S, Nasab M, Masihi S (2023). Effect of tele-medicine on health anxiety and pregnancy-related anxiety in pregnant women during the COVID-19 epidemic in Iran. Iran J Nurs Midwifery Res.

[83] Jannati N, Mazhari S, Ahmadian L, Mirzaee M (2020). Effectiveness of an app-based cognitive behavioral therapy program for postpartum depression in primary care: a randomized controlled trial. Int J Med Inform.

[84] Kanuri N, Arora P, Talluru S, Colaco B, Dutta R, Rawat A, Taylor BC, Manjula M, Newman MG (2020). Examining the initial usability, acceptability and feasibility of a digital mental health intervention for college students in India. Int J Psychol.

[85] Kola L, Abiona D, Oladeji BD, Ayinde O, Bello T, Gureje O (2022). Theory-driven development of a mobile phone supported intervention for adolescents with perinatal depression. Soc Psychiatry Psychiatr Epidemiol.

[86] Kusumadewi AF, Marchira CR, Widyandana D, Wirasto RT (2025). Randomized clinical trial comparing the effects of an asynchronous mobile application to guided brief cognitive behavioral therapy for managing anxiety among medical students. Trends Psychiatry Psychother.

[87] Lahti M, Groen G, Mwape L, Korhonen J, Breet E, Chapima F, Coetzee M, Ellilä H, Jansen R, Jonker D, Jörns-Presentati A, Mbanga I, Mukwato P, Mundenda J, Mutagubya J, Janse van Rensburg-Bonthuyzen E, Seedat S, Stein DJ, Suliman S, Sukwa T, Turunen T, Valtins K, van den Heuvel L, Wahila R, Grobler G (2020). Design and development process of a youth depression screening m-health application for primary health care workers in South Africa and Zambia: an overview of the MEGA project. Issues Ment Health Nurs.

[88] Logie C, Okumu M, Hakiza R, Kibuuka Musoke D, Berry I, Mwima S, Kyambadde P, Kiera UM, Loutet M, Neema S, Newby K, McNamee C, Baral SD, Lester R, Musinguzi J, Mbuagbaw L (2021). Mobile health-supported HIV self-testing strategy among urban refugee and displaced youth in Kampala, Uganda: protocol for a cluster randomized trial (Tushirikiane, supporting each other). JMIR Res Protoc.

[89] Luitel NP, Neupane V, Lamichhane B, Koirala GP, Gautam K, Karki E, Adhikari S, Votruba N, Jordans MJ, Kohrt BA, Carswell K, Thornicroft G, Lempp H (2023). Experience of primary healthcare workers in using the mobile app-based WHO mhGAP intervention guide in detection and treatment of people with mental disorders: a qualitative study in Nepal. SSM Ment Health.

[90] Maharjan SM, Poudyal A, van Heerden A, Byanjankar P, Thapa A, Islam C, Kohrt BA, Hagaman A (2021). Passive sensing on mobile devices to improve mental health services with adolescent and young mothers in low-resource settings: the role of families in feasibility and acceptability. BMC Med Inform Decis Mak.

[91] Mazhari S, Sabahi A, Gilanipour H, Keshvardoost S (2022). Agreement for diagnosis of depression and anxiety between self-assessment with e-questionnaire and psychiatric telephone interview among post-COVID-19 patients. J Educ Health Promot.

[92] Moulaei K, Bahaadinbeigy K, Mashoof E, Dinari F (2023). Design and development of a mobile-based self-care application for patients with depression and anxiety disorders. BMC Med Inform Decis Mak.

[93] Mukherjee A, Yatirajula SK, Kallakuri S, Paslawar S, Lempp H, Raman U, Essue BM, Sagar R, Singh R, Peiris D, Norton R, Thornicroft G, Maulik PK (2024). Using formative research to inform a mental health intervention for adolescents living in Indian slums: the ARTEMIS study. Child Adolesc Psychiatry Ment Health.

[94] Muniswamy P, Gorhe V, Parashivakumar L, Chandrasekaran B (2022). Short-term effects of a social media-based intervention on the physical and mental health of remotely working young software professionals: a randomised controlled trial. Appl Psychol Health Well Being.

[95] Nabunya P, Cavazos-Rehg P, Mugisha J, Kasson E, Namuyaba OI, Najjuuko C, Nsubuga E, Filiatreau LM, Mwebembezi A, Ssewamala FM (2024). An mHealth intervention to address depression and improve antiretroviral therapy adherence among youths living with HIV in Uganda: protocol for a pilot randomized controlled trial. JMIR Res Protoc.

[96] Najafi P, Hadizadeh M, Cheong JP, Mohafez H, Abdullah S, Poursadeghfard M (2023). Effects of tele-Pilates and tele-yoga on biochemicals, physical, and psychological parameters of females with multiple sclerosis. J Clin Med.

[97] Najafi P, Hadizadeh M, Cheong JP, Motl RW, Abdullah S, Mohafez H, Poursadeghfard M (2023). Effects of tele-exercise training on physical and mental health and quality of life in multiple sclerosis: do the effects differ by modality and clinical disease course?. Mult Scler Relat Disord.

[98] Naveen KH, Singh D, Srinivasan S, Bhardwaj P, Mitra P, Shukla KK, Tomo S, Dwivedi K, Sharma PP, Baskaran P, Shukla R, Vyas H, Kumar A, Midha N, Garg MK, Sharma P, Misra S (2024). Effect of tele-yoga on burnout, mental health and immune markers of health care workers on COVID-19 duty: an open-label parallel group pilot randomized controlled trial. Complement Ther Med.

[99] Njoroge W, Maina R, Frank E, Atwoli L, Wu Z, Ngugi AK, Sen S, Wang J, Wong S, Baker JA, Weinheimer-Haus EM, Khakali L, Aballa A, Orwa J, K Nyongesa M, Shah J, Waljee AK, Abubakar A, Merali Z (2023). Use of mobile technology to identify behavioral mechanisms linked to mental health outcomes in Kenya: protocol for development and validation of a predictive model. BMC Res Notes.

[100] Nurbaeti I, Syafii M, Lestari KB (2021). Developing an android-based application for early detection of postpartum depression symptoms in Indonesia. Belitung Nurs J.

[101] Nwaogu JM, Chan AP, Naslund JA, Hon CK, Belonwu C, Yang J (2021). Exploring the barriers to and motivators for using digital mental health interventions among construction personnel in Nigeria: qualitative study. JMIR Form Res.

[102] Ofoegbu TO, Asogwa U, Otu MS, Ibenegbu C, Muhammed A, Eze B (2020). Efficacy of guided internet-assisted intervention on depression reduction among educational technology students of Nigerian universities. Medicine (Baltimore).

[103] Pasyar N, Sam A, Rivaz M, Nazarinia M (2023). A smartphone-based supportive counseling on health anxiety and acceptance of disability in Systemic Lupus Erythematosus patients: a randomized clinical trial. Patient Educ Couns.

[104] Patel VL, Halpern M, Nagaraj V, Chang O, Iyengar S, May W (2021). Information processing by community health nurses using mobile health (mHealth) tools for early identification of suicide and depression risks in Fiji Islands. BMJ Health Care Inform.

[105] Pozuelo JR, Moffett BD, Davis M, Stein A, Cohen H, Craske MG, Maritze M, Makhubela P, Nabulumba C, Sikoti D, Kahn K, Sodi T, van Heerden A, O'Mahen HA (2023). User-centered design of a gamified mental health app for adolescents in sub-Saharan Africa: multicycle usability testing study. JMIR Form Res.

[106] Putri AF, Marsetyo FA, Atmaja US, Fuad A (2022). Mental health teleconsultation patterns on the most popular mHealth platforms in Indonesia. Stud Health Technol Inform.

[107] Rabbani F, Nafis J, Akhtar S, Khan MS, Sayani S, Siddiqui A, Siddiqi S, Merali Z (2024). Technology-assisted mental health intervention delivered by frontline workers at community doorsteps for reducing anxiety and depression in rural Pakistan: protocol for the mPareshan mixed methods implementation trial. JMIR Res Protoc.

[108] Ramnath S, Suri G (2021). Managing depression in India: opportunities for a targeted smartphone app. Int J Soc Psychiatry.

[109] Roshid MM, Rahman MM, Alam MN, Banu B, Eity KF, Shahin RR, Shaba S, Rahman M, Sarker M, Okamura H (2024). Combined effects of the mobile health (mHealth) psychoeducation and Benson relaxation technique in reducing the caregiving burden of cancer patients in Bangladesh: a protocol for a randomized controlled trial. Cureus.

[110] Safaei M, Mahdavi A, Mehdipour-Rabori R (2024). Designing and evaluating a mobile app to assist patients undergoing coronary angiography and assessing its impact on anxiety, stress levels, and self-care. BMC Med Inform Decis Mak.

[111] Saleem M, Zafar S, Klein T, Koesters M, Bashir A, Fuhr DC, Sikander S, Zeeb H (2025). A digital tool (technology-assisted problem management plus) for lay health workers to address common mental health disorders: co-production and usability study in Pakistan. JMIR Form Res.

[112] Salisbury TT, Kohrt BA, Bakolis I, Jordans MJ, Hull L, Luitel NP, McCrone P, Sevdalis N, Pokhrel P, Carswell K, Ojagbemi A, Green EP, Chowdhary N, Kola L, Lempp H, Dua T, Milenova M, Gureje O, Thornicroft G (2021). Adaptation of the World Health Organization electronic mental health gap action programme intervention guide app for mobile devices in Nepal and Nigeria: protocol for a feasibility cluster randomized controlled trial. JMIR Res Protoc.

[113] Seddigh S, Bagheri S, Sharifi N, Moravej H, Hadian Shirazi Zahra (2023). The effect of yoga therapy directed by virtual training on depression of adolescent girls with type 1 diabetes: a randomized controlled trial. J Diabetes Metab Disord.

[114] Setiawan H, Xu W, Wang C, Li C, Ariyanto H, Firdaus FA, Mustopa AH, Hidayat N, Hu R (2025). The effect of mobile application based genetic counseling on the psychosocial well-being of thalassemia patients and caregivers: a randomized controlled trial. Patient Educ Couns.

[115] Shania M, Handayani PW, Asih S (2023). Designing high-fidelity mobile health for depression in Indonesian adolescents using design science research: mixed method approaches. JMIR Form Res.

[116] Shaygan M, Yazdani Z, Rambod M (2023). The effect of interactive virtual psycho-educational interventions via social networks on self-efficacy and anxiety among patients infected with COVID-19 and living in home quarantine: a randomized control trial. Iran J Nurs Midwifery Res.

[117] Singla DR, Fernandes L, Savel K, Shah A, Agrawal R, Bhan A, Nadkarni A, Sharma A, Khan A, Lahiri A, Tugnawat D, Lesh N, Patel V, Naslund J (2024). Erratum: scaling up the task-sharing of psychological therapies: a formative study of the PEERS smartphone application for supervision and quality assurance in rural India - CORRIGENDUM. Glob Ment Health (Camb).

[118] Sinha C, Meheli S, Kadaba M (2023). Understanding digital mental health needs and usage with an artificial intelligence-led mental health app (Wysa) during the COVID-19 pandemic: retrospective analysis. JMIR Form Res.

[119] Soltani Z, Parizad N, Radfar M, Alinejad V, Arzanlo M, Haghighi M (2024). The effect of the Yara smartphone application on anxiety, sleep quality, and suicidal thoughts in patients with major depressive disorder in Iran: a randomized controlled trial. BMC Psychiatry.

[120] Than TN, Nguyen TT, Nguyen TC, Vu LT, Vo PT, TTruong K, Schofield P, Nguyen TA (2023). Smartphone app-based intervention for reducing stress, depression, and anxiety in caregivers of people with dementia in Vietnam: study protocol for a pilot randomized controlled trial. Digit Health.

[121] Torrey WC, Cepeda M, Castro S, Bartels SM, Cubillos L, Obando FS, Camblor PM, Uribe-Restrepo JM, Williams M, Gómez-Restrepo C, Marsch LA (2020). Implementing technology-supported care for depression and alcohol use disorder in primary care in Colombia: preliminary findings. Psychiatr Serv.

[122] van Heerden A, Poudyal A, Hagaman A, Maharjan SM, Byanjankar P, Bemme D, Thapa A, Kohrt BA (2024). Integration of passive sensing technology to enhance delivery of psychological interventions for mothers with depression: the StandStrong study. Sci Rep.

[123] Vera Cruz Dos Santos D, Coelho de Soárez P, Cavero V, U Rocha TI, Aschar S, Daley K, Garcia Claro H, Abud Scotton G, Fernandes I, Diez-Canseco F, Brandt L, Toyama M, Martins Castro HC, Miranda JJ, Araya R, Quayle J, Rossi Menezes P (2021). A mobile health intervention for patients with depressive symptoms: protocol for an economic evaluation alongside two randomized trials in Brazil and Peru. JMIR Res Protoc.

[124] Watanabe K, Tran TT, Sripo N, Sakuraya A, Imamura K, Boonyamalik P, Sasaki N, Tienthong T, Asaoka H, Iida M, Nguyen QT, Nguyen NT, Vu ST, Ngo TT, Luyen TT, Nguyen LD, Nguyen NT, Nguyen BT, Matsuyama Y, Takemura Y, Nishi D, Tsutsumi A, Nguyen HT, Kaewboonchoo O, Kawakami N (2024). Effectiveness of a smartphone-based stress management program for depression in hospital nurses during COVID-19 in Vietnam and Thailand: 2-arm parallel-group randomized controlled trial. J Med Internet Res.

[125] Weierstall R, Crombach A, Nandi C, Bambonyé Manassé, Probst T, Pryss R (2021). Effective adoption of tablets for psychodiagnostic assessments in rural Burundi: evidence for the usability and validity of mobile technology in the example of differentiating symptom profiles in AMISOM soldiers 1 year after deployment. Front Public Health.

[126] Zamanifard M, Soltanian M, Edraki M, Moravaj H, Sharifi N (2022). The effects of virtual directed painting therapy on anxiety, depression, and self-efficacy of children with type 1 diabetes: a randomized controlled clinical trial. Int J Community Based Nurs Midwifery.

[127] Zuccolo PF, Xavier MO, Matijasevich A, Polanczyk G, Fatori D (2021). A smartphone-assisted brief online cognitive-behavioral intervention for pregnant women with depression: a study protocol of a randomized controlled trial. Trials.

[128] Patterson VC, Rossi MA, Pencer A, Wozney L (2022). An internet-based cognitive behavioral therapy program for anxiety and depression (tranquility): adaptation co-design and fidelity evaluation study. JMIR Form Res.

[129] Greenhalgh T, Robert G, Macfarlane F, Bate P, Kyriakidou O (2004). Diffusion of innovations in service organizations: systematic review and recommendations. Milbank Q.

[130] Pérez D, Van der Stuyft P, Zabala MC, Castro M, Lefèvre P (2016). A modified theoretical framework to assess implementation fidelity of adaptive public health interventions. Implement Sci.

[131] Bonar JR, Wright S, Yadrich DM, Werkowitch M, Ridder L, Spaulding R, Smith CE (2020). Maintaining intervention fidelity when using technology delivery across studies. Comput Inform Nurs.

[132] Arean PA, Hallgren KA, Jordan JT, Gazzaley A, Atkins DC, Heagerty PJ, Anguera JA (2016). The use and effectiveness of mobile apps for depression: results from a fully remote clinical trial. J Med Internet Res.

[133] Molloy A, Anderson PL (2021). Engagement with mobile health interventions for depression: a systematic review. Internet Interv.

[134] Connolly SL, Hogan TP, Shimada SL, Miller CJ (2021). Leveraging implementation science to understand factors influencing sustained use of mental health apps: a narrative review. J Technol Behav Sci.

[135] Stawarz K, Preist C, Tallon D, Wiles N, Coyle D (2018). User experience of cognitive behavioral therapy apps for depression: an analysis of app functionality and user reviews. J Med Internet Res.

[136] Chan AH, Honey ML (2022). User perceptions of mobile digital apps for mental health: acceptability and usability - An integrative review. J Psychiatr Ment Health Nurs.

[137] Abd-Alrazaq A, Safi Z, Alajlani M, Warren J, Househ M, Denecke K (2020). Technical metrics used to evaluate health care chatbots: scoping review. J Med Internet Res.

[138] Maramba I, Chatterjee A, Newman C (2019). Methods of usability testing in the development of eHealth applications: a scoping review. Int J Med Inform.

[139] Khademian F, Aslani A, Bastani P (2020). The effects of mobile apps on stress, anxiety, and depression: overview of systematic reviews. Int J Technol Assess Health Care.

[140] Boucher EM, Raiker JS (2024). Engagement and retention in digital mental health interventions: a narrative review. BMC Digit Health.

[141] Borghouts J, Eikey E, Mark G, De Leon C, Schueller SM, Schneider M, Stadnick N, Zheng K, Mukamel D, Sorkin DH (2021). Barriers to and facilitators of user engagement with digital mental health interventions: systematic review. J Med Internet Res.

[142] Lyles CR, Sarkar U, Osborn CY (2014). Getting a technology-based diabetes intervention ready for prime time: a review of usability testing studies. Curr Diab Rep.

[143] O'Brien HL, Toms EG (2008). What is user engagement? A conceptual framework for defining user engagement with technology. J Am Soc Inf Sci.

[144] Scheuer L, Torous J (2022). Usable data visualization for digital biomarkers: an analysis of usability, data sharing, and clinician contact. Digit Biomark.

[145] Mustafa AS, Ali N, Dhillon JS, Alkawsi G, Baashar Y (2022). User engagement and abandonment of mHealth: a cross-sectional survey. Healthcare (Basel).

[146] Nadal C, Sas C, Doherty G (2020). Technology acceptance in mobile health: scoping review of definitions, models, and measurement. J Med Internet Res.

[147] Perski O, Short CE (2021). Acceptability of digital health interventions: embracing the complexity. Transl Behav Med.

[148] Hall GC, Ibaraki AY, Huang ER, Marti CN, Stice E (2016). A meta-analysis of cultural adaptations of psychological interventions. Behav Ther.

[149] Shehadeh MH, Heim E, Chowdhary N, Maercker A, Albanese E (2016). Cultural adaptation of minimally guided interventions for common mental disorders: a systematic review and meta-analysis. JMIR Ment Health.

[150] Kirmayer LJ, Jarvis GE (2019). Culturally responsive services as a path to equity in mental healthcare. Healthc Pap.

[151] Kirmayer LJ, Narasiah L, Munoz M, Rashid M, Ryder AG, Guzder J, Hassan G, Rousseau C, Pottie K, Canadian Collaboration for ImmigrantRefugee Health (CCIRH) (2011). Common mental health problems in immigrants and refugees: general approach in primary care. CMAJ.

[152] Arora PG, Parr KM, Khoo O, Lim K, Coriano V, Baker CN (2021). Cultural adaptations to youth mental health interventions: a systematic review. J Child Fam Stud.

[153] Naderbagi A, Loblay V, Zahed IU, Ekambareshwar M, Poulsen A, Song YJ, Ospina-Pinillos L, Krausz M, Mamdouh Kamel M, Hickie IB, LaMonica HM (2024). Cultural and contextual adaptation of digital health interventions: narrative review. J Med Internet Res.

[154] Brian RM, Ben-Zeev D (2014). Mobile health (mHealth) for mental health in Asia: objectives, strategies, and limitations. Asian J Psychiatr.

[155] Liem A, Natari RB, Hall BJ, Jimmy (2021). Digital health applications in mental health care for immigrants and refugees: a rapid review. Telemed J E Health.

[156] Lim Q Framing the issues: digital mental health in Asia. Milken Institute.

[157] Murphy JK, Khan A, Sun Q, Minas H, Hatcher S, Ng CH, Withers M, Greenshaw A, Michalak EE, Chakraborty PA, Sandanasamy KS, Ibrahim N, Ravindran A, Chen J, Nguyen VC, Lam RW (2021). Needs, gaps and opportunities for standard and e-mental health care among at-risk populations in the Asia Pacific in the context of COVID-19: a rapid scoping review. Int J Equity Health.

[158] Musoke D, Nyashanu M, Bugembe H, Lubega GB, O'Donovan J, Halage AA, Gibson L (2022). Contested notions of challenges affecting community health workers in low- and middle-income countries informed by the silences framework. Hum Resour Health.

[159] Watts S, Mackenzie A, Thomas C, Griskaitis A, Mewton L, Williams A, Andrews G (2013). CBT for depression: a pilot RCT comparing mobile phone vs. computer. BMC Psychiatry.

[160] Leuchtenberg S, Gromer D, Käthner I (2022). Videoconferencing versus face-to-face psychotherapy: Insights from patients and psychotherapists about comparability of therapeutic alliance, empathy and treatment characteristics. Couns Psychother Res.

[161] Ierardi E, Bottini M, Riva Crugnola C (2022). Effectiveness of an online versus face-to-face psychodynamic counselling intervention for university students before and during the COVID-19 period. BMC Psychol.

[162] Cataldo F, Chang S, Mendoza A, Buchanan G (2021). A perspective on client-psychologist relationships in videoconferencing psychotherapy: literature review. JMIR Ment Health.

[163] Newman MG, Szkodny LE, Llera SJ, Przeworski A (2011). A review of technology-assisted self-help and minimal contact therapies for anxiety and depression: is human contact necessary for therapeutic efficacy?. Clin Psychol Rev.

[164] Donker T, Kleiboer A (2018). Special issue: e-health innovations for global mental health. Glob Ment Health (Camb).

[165] Clay RA Mental health apps are gaining traction. American Psychological Association.

[166] Curry D, Islam MA, Sarker BK, Laterra A, Khandaker I (2023). A novel approach to frontline health worker support: a case study in increasing social power among private, fee-for-service birthing attendants in rural Bangladesh. Hum Resour Health.

[167] Paxling B, Lundgren S, Norman A, Almlöv J, Carlbring P, Cuijpers P, Andersson G (2013). Therapist behaviours in internet-delivered cognitive behaviour therapy: analyses of e-mail correspondence in the treatment of generalized anxiety disorder. Behav Cogn Psychother.

[168] Lee EE, Torous J, De Choudhury M, Depp CA, Graham SA, Kim H, Paulus MP, Krystal JH, Jeste DV (2021). Artificial intelligence for mental health care: clinical applications, barriers, facilitators, and artificial wisdom. Biol Psychiatry Cogn Neurosci Neuroimaging.

[169] Camacho E, Cohen A, Torous J (2022). Assessment of mental health services available through smartphone apps. JAMA Netw Open.

[170] Olawade DB, Wada OZ, Odetayo A, David-Olawade AC, Asaolu F, Eberhardt J (2024). Enhancing mental health with artificial intelligence: current trends and future prospects. J Med Surg Public Health.

[171] Saeidnia HR, Hashemi Fotami SG, Lund B, Ghiasi N (2024). Ethical considerations in artificial intelligence interventions for mental health and well-being: ensuring responsible implementation and impact. Soc Sci.

