# Supplementary material for: Digital Health Interventions for Depression and Anxiety in Low- and Middle-Income Countries: Rapid Scoping Review
Source: JMIR Ment Health. 2025 Aug 22;12:e68296. doi: 10.2196/68296 (PMC12413575; doi:10.2196/68296)
Supplement: Multimedia Appendix 1 [file mental_v12i1e68296_app1.docx]

### Table S1: Final PubMed search strategy

| Search number | Query | Results  (March 13, 2025) |
| --- | --- | --- |
| 1 | (smartphone[MeSH]) OR (“mobile applications”[MeSH]) OR (telemedicine[MeSH]) OR (telehealth[MeSH]) OR (“mobile health”[MeSH]) | 69,860 |
| 2 | **("digital technology"[Text Word]) OR ("smartphone app*"[Text Word]) OR (telehealth[Text Word]) OR (telemedicine[Text Word]) OR ("mobile app*"[Text Word]) OR ("digital health"[Text Word]) OR (ehealth[Text Word]) OR (e-health[Text Word]) OR (“electronic health”[Text Word] OR (mhealth[Text Word]) OR (m-health[Text Word]) OR (“mobile health”[Text Word]) OR (virtual[Text Word]) OR (tele*[Text Word])** | 475,060 |
| 3 | #1 or #2 | 481,949 |
| 4 | **("mood disorders"[**MeSH**]) OR (“depressive disorders”[**MeSH**]) OR ("anxiety disorders"[**MeSH**])** | 253,142 |
|  | **(d**epress*[Text Word]) OR (anxi*[Text Word]) | 897,194 |
| 6 | #4 or #5 | 960,767 |
| 7 | **("developing countries"[**MeSH**])** | 83,671 |
| 8[1] | (MH "Low and Middle Income Countries")  OR (MH "Honduras") OR (MH "Nicaragua") OR (MH "Belize") OR (MH "El Salvador") OR (MH "Bolivia") OR (MH "Venezuela") OR (MH "Haiti") OR (MH "Tajikistan") OR (MH "Kyrgyzstan") OR (MH "Myanmar") OR (MH "Vietnam") OR (MH "Philippines") OR (MH "Indonesia") OR (MH "East Timor") OR (MH "Cambodia") OR (MH "Bhutan") OR (MH "Pakistan") OR (MH "Nepal") OR (MH "Sri Lanka") OR (MH "Afghanistan") OR (MH "Iran") OR (MH "Syria") OR (MH "Yemen") OR (MH "Mongolia") OR (MH "Low and Middle Income Countries") OR (MH "Algeria") OR (MH "Egypt") OR (MH "Morocco") OR (MH "Tunisia") OR (MH "Cameroon") OR (MH "Burundi") OR (MH "Central African Republic") OR (MH "Chad") OR (MH "Democratic Republic of the Congo") OR (MH "Equatorial Guinea") OR (MH "Congo") OR (MH "Djibouti") OR (MH "Eritrea") OR (MH "Ethiopia") OR (MH "Kenya") OR (MH "Rwanda") OR (MH "Somalia") OR (MH "Sudan") OR (MH "Tanzania") OR (MH "Angola") OR (MH "Zimbabwe") OR (MH "Malawi") OR (MH "Mozambique") OR (MH "Lesotho") OR (MH "Zambia") OR (MH "Benin") OR (MH "Burkina Faso") OR (MH "Cape Verde") OR (MH "Cote d'Ivoire") OR (MH "Gambia") OR (MH "Ghana") OR (MH "Guinea") OR (MH "Guinea-Bissau") OR (MH "Liberia") OR (MH "Mali") OR (MH "Mauritania") OR (MH "Niger") OR (MH "Nigeria") OR (MH "Senegal") OR (MH "Togo") OR (MH "Sierra Leone") OR (MH "Ukraine") OR (MH "India") OR (MH "Bangladesh") OR (MH "Laos") or (LMIC or LMICs or (low* N3 (income countr* or income nation*)) or Afghanistan OR Afghani  OR Algeria*  OR Angola* OR "Guinea-Bissau" OR Somalia OR Bangladesh* OR Belize OR Benin OR Bhutan or Bhutanese OR Bolivia* OR "Burkina Faso" OR Burundi OR "Central African Republic" OR "Cabo Verde" OR "Cape Verde" or Cambodia*  OR Cameroon OR Chad OR Tchad OR Comoros OR Congo OR "Cote d Ivoire" OR "Ivory Coast" OR Djibouti OR Egypt*  OR "El Salvador" OR Salvadoran OR Eritrea* OR Eswatini  OR Ethiopia*  OR Gambia OR Gaza OR Ghana OR Guinea OR Haiti OR Honduras* OR India  OR Indonesia OR Iran OR Kenya or Kenyan OR Kiribati OR Kyrgyz OR Laos OR Lesotho OR Liberia  OR Madagascar  OR Malawi  OR Mali OR Mauritania OR Micronesia  OR Mongolia OR Morocco OR Mozambique OR Myanmar OR  Nepal OR Nepalese OR Nicaragua* OR Niger OR Nigerien OR Nigeria* OR "North Korea" OR  "Papua New Guinea" OR Pakistan OR Philippines OR Principe  OR "Sierra Leone" OR "South Sudan" OR Sudan OR Syria OR Syrian OR Rwanda* OR Samoa* OR "Sao Tome" OR Senegal* OR "Solomon Islands"OR "Sri Lanka" OR Tanzania OR Tajikistan OR "Timor-Leste" OR Togo OR Tunisia OR Uganda OR Ukraine OR Uzbekistan  OR Vanuatu OR Vietnam  OR "West Bank" OR Yemen  OR Zambia OR Zimbabwe) | 2,521,485 |
| 9 | **#7 OR #8** | 2,521,485 |
| 10 | #3 AND #6 AND #9 | 1,385 |
| 11 | Limit to January 1, 2020 – January 31, 2025 | 998 |
| 12 | Limit to English language | 995 |

### References

1. Geoffrey & Robyn Sperber Health Sciences Library U of A. A filter to identify countries with low or lower middle income economies in the MEDLINE database. 2021.
