# Supplementary material for: Digital Health Interventions for Depression and Anxiety in Low- and Middle-Income Countries: Rapid Scoping Review
Source: JMIR Ment Health. 2025 Aug 22;12:e68296. doi: 10.2196/68296 (PMC12413575; doi:10.2196/68296)
Supplement: Multimedia Appendix 2 [file mental_v12i1e68296_app2.docx]

### Table S2: Final PsycINFO search strategy

| Search number | Query | Results  (March 13, 2025) |
| --- | --- | --- |
| 1 | MA smartphone OR MA "mobile applications" OR MA telemedicine OR MA telehealth OR MA "mobile health" | 5,953 |
| 2 | KW "digital technology" OR KW "smartphone app" OR KW telehealth OR KW telemedicine OR KW "mobile app" OR KW "digital health" OR KW ehealth OR KW e-health OR KW "electronic health" OR KW mhealth OR KW m-health OR KW "mobile health" | 14,475 |
| 3 | KW virtual OR KW tele* | 43,621 |
| 4 | #1 or #2 or #3 | 53,988 |
| 5 | MA "mood disorders" OR MA "depressive disorder" OR MA "anxiety disorders" | 80,925 |
| 6 | KW depress* OR KW anxi* | 282,108 |
| 7 | #5 or #6 | 307,189 |
| 8 | MA "developing countries" | 4,398 |
| 9[1] | (angola/ or benin/ or "burkina faso"/ or burundi/ or cameroon/ or "cape verde"/ or "central african republic"/ or chad/ or comoros/ or congo/ or "cote d'ivoire"/ or "democratic republic congo"/ or djibouti/ or "equatorial guinea"/ or eritrea/ or ethiopia/ or gabon/ or gambia/ or ghana/ or guinea/ or guinea-bissau/ or kenya/ or lesotho/ or liberia/ or madagascar/ or malawi/ or mali/ or mozambique/ or namibia/ or niger/ or nigeria/ or rwanda/ or senegal/ or "sierra leone"/ or exp somalia/ or "south sudan"/ or sudan/ or swaziland/ or tanzania/ or togo/ or uganda/ or zimbabwe/ or algeria/ or egypt/ or mauritania/ or morocco/ or tunisia/ or "el salvador"/ or honduras/ or nicaragua/ or bolivia/ or "syrian arab republic"/ or yemen/ or haiti/ or exp "georgia (republic)"/ or kosovo/ or Mongolia/ or "sao tome and principe"/ or exp ukraine/ or afghanistan/ or bangladesh/ or bhutan/ or exp india/ or exp Iran/ or nepal/ or exp pakistan/ or "sri lanka"/ or kyrgyzstan/ or tajikistan/ or uzbekistan/ or cambodia/ or laos/ or myanmar/ or "papua new guinea"/ or timor-leste/ or "viet nam"/ or "federated states of micronesia"/ or kiribati/ or philippines/ or samoa/ or "solomon islands"/ or vanuatu/ or "North Korea"/ or "sao tome and principe"/ or ((low* adj3 (income countr* or income nation*)) or LMIC or LMICs or Afghanistan or Afghani or Afghan or Angola* or Bangladesh* or Belize or Benin or Beninese or Bhutan or Bolivia* or "Burkina Faso" or Burkinabe or Burundi* or "Cabo Verde" or "Cape Verde" or Cambodia* or Cameroon* or "Central African Republic" or Chad or Chadian or Tchad or Comoros or Comoran or Congo or Congolese or "Cote d'ivoire" or Ivorian or Djibouti or Egypt or Egyptian or "El Salvador" or Salvadoran or Eritrea* or Ethiopia* or Gambia or Gambian or (Georgia not "United States") or Ghana* or Guinea or "Guinea Bissau*" or Haiti or Haitian or Hondura* or India or (Indian not American) or Indonesia* or Kazakhstan or Kenya* or Kiribati or "North Korea*" or DPRK or Kosovo or Kosovar or Kosovan or Kyrgyz* or Laos or Laotian or Lesotho or Mosotho or Basotho or Liberia* or Madagascar or Malagasy or Malawi* or Mali or Malian or Mauritania* or Micronesia* or Moldova* or Mongolia* or Morocco or Moroccan or Mozambique or Mozambican or Myanmar or Burmese or Myanmarese or Nepal or Nepalese or Nicaragua* or Niger or Nigerien* or Nigeria* or Pakistan* or "Papua New Guinea*" or Philippines or Filipino* or Rwanda* or Samoa or "Sao Tome and Principe" or "San Tomean" or Senegal* or "Sierra Leone*" or "Solomon Island*" or Somalia* or "Sri Lanka*" or Sudan or Sudanese or Swaziland or Swazi or Syria or Syrian or Tajikistan or Tajik or Tadzhik or Tanzania* or "Timor Leste" or Timorese or Togo or Togolese or Tunisia* or Uganda* or Ukraine or Ukrainian or Uzbekistan* or Uzbeki or Vanuatu or Vietnam* or "West Bank" or Gaza or Yemen* or Zambia* or Zimbabwe*).tw,cp.) | 88,139 |
| 10 | **#8 OR #9** | 91,176 |
| 11 | #4 AND #7 AND #10 | 28 |
| 12 | Limit to January 1, 2020 – January 31, 2025 | 15 |
| 13 | Limit to English language | 15 |

### References

1. Geoffrey & Robyn Sperber Health Sciences Library U of A. A filter to identify countries with low or lower middle income economies in the MEDLINE database. 2021.
