# Supplementary material for: Digital Health Interventions for Depression and Anxiety in Low- and Middle-Income Countries: Rapid Scoping Review
Source: JMIR Ment Health. 2025 Aug 22;12:e68296. doi: 10.2196/68296 (PMC12413575; doi:10.2196/68296)
Supplement: Multimedia Appendix 4 [file mental_v12i1e68296_app4.docx]

### Table S3: Summary of the excluded articles after full-text review

| **Author (year)** | **Article Title** | **Reason for Exclusion** |
| --- | --- | --- |
| Adiukwu et al. (2022) [1] | Mental health distress during the COVID-19 pandemic in Nigeria: Need for psychological intervention. | Not a DMHI |
|  |  |  |
| Aga et al. (2023) [2] | Employing telepsychiatry services to assess the prevalence and identify mental health disorders using the PHQ-9 and GAD-7 in resource-constrained regions of Dadar Mansehra, Pakistan: An observational cross-sectional study. | Not a DMHI |
|  |  |  |
| Ahmed et al. (2021) [3] | Willingness to use telemedicine during COVID-19 among health professionals in a low income country. | Focus is on another topic |
|  |  |  |
| Ahmed et al. (2024) [4] | Investigating rhythmicity in app usage to predict depressive symptoms: Protocol for personalized framework development and validation through a countrywide study. | Focus is on another topic |
|  |  |  |
| Ajmera et al. (2023) [5] | Impact of telehealth interventions on physiological and psychological outcomes in breast cancer survivors: A meta-analysis of randomised controlled trials. | Focus is global |
|  |  |  |
| Akingbade et al. (2023) [6] | Effect of mHealth interventions on psychological issues experienced by women undergoing chemotherapy for breast cancer: A systematic review and meta-analysis. | Focus is global |
|  |  |  |
| Alam et al. (2022) [7] | Comparison of the young aged and elderly female users' adoption of mHealth services. | Focus is on mental health in general |
|  |  |  |
| Ali et al. (2020) [8] | Leveraging the use of mHealth in patients with depression  among low- and middle-income countries. | Does not describe use of a DMHI |
|  |  |  |
| AlQarni et al. (2023) [9] | Brief tele-mindfulness-based intervention: A multicenter randomized controlled trial | Focus is not on LMICs |
|  |  |  |
| Andersen et al. (2023) [10] | Virtual reality interventions for the treatment of anxiety disorders: A scoping review. | Focus is global |
|  |  |  |
| Angwenyi et al. (2024) [11] | Engaging fathers(to-be): A pilot study on the adaptation and programme experience of SMS4baba intervention in Kenya’s informal settlements. | Focus is on other topics |
|  |  |  |
| Ansaari et al. (2024) [12] | Efficacy of in-person versus digital mental health interventions for postpartum depression: Meta-analysis of randomized controlled trials. | Focus is global |
|  |  |  |
| Askarizadeh et al. (2025) [13] | Determining the impact of mobile-based self-care applications on reducing anxiety in healthcare providers: a systematic review. | Focus is not on LMICs |
|  |  |  |
| Atiq et al. (2024) [14] | Exploring preference for delivery methods for a psychosocial intervention for prenatal anxiety: A qualitative study from a tertiary care hospital in Pakistan. | Not a DMHI |
|  |  |  |
| Babaei et al. (2023) [15] | The effect of virtual reminiscence therapy on depression and anxiety in patients with gastric cancer undergoing chemotherapy. | Not a DMHI |
|  |  |  |
| Baradwan et al. (2025) [16] | The impact of virtual reality on maternal anxiety, satisfaction, and fetal outcomes among pregnant women undergoing non-stress tests: A systematic review and meta-analysis of randomized controlled trials | Focus is not on LMICs |
|  |  |  |
| Beatty et al. (2022) [17] | Evaluating the therapeutic alliance with a free-text CBT conversational agent (Wysa): A mixed-methods study. | No location specified |
|  |  |  |
| Ben-Zeev et al. (2021) [18] | Smartphone intervention for people with serious mental illness: Fully remote randomized controlled trial of CORE. | Focus is on mental health in general |
|  |  |  |
| Beniwal et al. (2020) [19] | Protocol of a multi-centric randomized controlled trial to evaluate efficacy of telephone-based psychosocial interventions on future suicide risk in suicide attempters. | Primary focus is not depression or anxiety |
|  |  |  |
| Bhardwaj et al. (2024) [20] | Engaging husbands in a digital mental health intervention to provide tailored counseling for women experiencing postpartum depression: A mixed methods study in Nepal | Focus is on other topics |
|  |  |  |
| Bhat et al. (2020) [21] | Can mobile health improve depression treatment access and adherence among rural Indian women? A qualitative study. | Does not describe use of a DMHI |
|  |  |  |
| Bhatia et al. (2021) [22] | Increased risk of mental health disorders in patients with RA during the COVID-19 pandemic: A possible surge and solutions. | Focus is global |
|  |  |  |
| Bikmoradi et al. (2023) [23] | The impact of telenursing on level of depression, stress and anxiety in discharged patients after coronary artery bypass graft surgery: A randomized clinical trial. | Not a DMHI |
|  |  |  |
| Bosqui et al. (2023) [24] | What drives change in children receiving telephone- delivered Common Elements Treatment Approach (t-CETA)? A multiple n = 1 study with Syrian refugee children and adolescents in Lebanon. | Not a DMHI |
|  |  |  |
| Bui et al. (2022) [25] | eHealth interventions for the informal caregivers of people with dementia: A systematic review of systematic reviews. | Focus is global |
|  |  |  |
| Campisi et al. (2022) [26] | Mental health interventions in adolescence. | Does not describe use of a DMHI |
|  |  |  |
| Carter et al. (2021) [27] | The emergence of digital mental health in low-income and middle-income countries: A review of recent advances and implications for the treatment and prevention of mental disorders. | Focus is on mental health in general |
|  |  |  |
| Chakeri et al. (2020) [28] | Evaluating the effect of nurse-led telephone follow-ups (tele-nursing) on the anxiety levels in people with coronavirus. | Not a DMHI |
|  |  |  |
| Dantes et al. (2024) [29] | Brief mobile app–based mindfulness intervention for Indonesian senior high school teachers: Protocol for a pilot randomized controlled trial | Primary focus is not depression or anxiety |
|  |  |  |
| Dergaa et al. (2024) [30] | ChatGPT is not ready yet for use in providing mental health assessment and interventions. | Focus is on another topic |
|  |  |  |
| Dikaios et al. (2020) [31] | Connecting during COVID-19: A protocol of a volunteer-based telehealth program for supporting older adults' health | Focus is on mental health in general |
|  |  |  |
| Dixit et al. (2020) [32] | Promoting healthy lifestyles using information technology during the COVID-19 pandemic. | Focus is on mental health in general |
|  |  |  |
| Drissi et al. (2020) [33] | An analysis on self-management and treatment-related functionality and characteristics of highly  rated anxiety apps. | No location specified |
|  |  |  |
| Dyer et al. (2021) [34] | The psychosocial effects of the COVID-19 pandemic on youth living with HIV in Western Kenya. | Does not describe use of a DMHI |
|  |  |  |
| Dzinamarira et al. (2024) [35] | COVID-19 and mental health services in Sub-Saharan Africa: A critical literature review. | Not a DMHI |
|  |  |  |
| El-Refaay et al. (2024) [36] | Efficacy of technology-based mental health interventions in minimizing mental health symptoms among in immigrants, asylum seekers or refugees; systematic review | Focus is not on LMICs |
|  |  |  |
| Escrivá-Martínez et al. (2022) [37] | mHealth intervention to improve quality of life in patients with chronic diseases during the COVID-19 crisis in Paraguay: A study protocol for a randomized controlled trial. | Primary focus is not depression or anxiety |
|  |  |  |
| Farahimanesh et al. (2023) [38] | Effectiveness of a virtual-reality-based self-help intervention for lowering the psychological burden during the COVID-19 pandemic: Results from a randomized controlled trial in Iran | Focus is on mental health in general |
|  |  |  |
| Farzandipour et al. (2024) [39] | Effects of mhealth applications on military personnel's physical and mental health: A systematic review. | Focus is global |
|  |  |  |
| Fereidooni et al. (2024) [40] | Application of virtual reality for supportive care in cancer patients: A systematic review | Focus is on other topics |
|  |  |  |
| Feroz et al. (2021) [41] | Equipping community health workers with digital tools for pandemic response in LMICs. | Does not describe use of DMHI |
|  |  |  |
| Ganesan et al. (2023) [42] | Effects of tele-counseling on reducing anxiety leevels of COVID-19 patients in isolation wards: An observational study. | Not a DMHI |
|  |  |  |
| Garg et al. (2022) [43] | Integrating assisted tele-psychiatry into primary healthcare in Goa, India: a feasibility study. | Not a DMHI |
|  |  |  |
| Gautam et al. (2024) [44] | Preferences for mHealth intervention to address mental  health challenges among men who have sex with men in Nepal: Qualitative study. | Does not describe use of a DMHI |
|  |  |  |
| Gautama et al. (2023) [45] | Efficacy of smartphone-based virtual reality relaxation in providing comfort to patients with cancer undergoing chemotherapy in oncology outpatient setting in Indonesia: Protocol for a randomised controlled trial. | Primary focus is not depression or anxiety |
|  |  |  |
| Getu et al. (2023) [46] | The effect of cognitive behavioural therapy integrated with activity pacing on cancer-related fatigue, depression and quality of life among patients with breast cancer undergoing chemotherapy in Ethiopia: A randomised clinical trial. | Primary focus is not depression or anxiety |
|  |  |  |
| Gevers-Montoro et al. (2022) [47] | From hands-on to remote: Moderators of response to a novel self- management telehealth programme during the COVID-19 pandemic. | Focus is on another topic |
|  |  |  |
| Ghazanfarpour et al. (2022) [48] | Investigating the effectiveness of tele-counseling for the mental health of staff in hospitals and COVID-19 clinics: a clinical control trial. | Not a DMHI |
|  |  |  |
| Ghobadi et al. (2024) [49] | The effect of virtual reality on reducing patients' anxiety and pain during dental implant surgery. | Focus is on another topic |
|  |  |  |
| Grande et al. (2020) [50] | Effective/cost effective interventions of child mental health problems in low- and middle-income countries (LAMIC): Systematic review. | Focus is on mental health in general |
|  |  |  |
| Guo et al. (2024) [51] | Cognitive-behavioral treatment for insomnia and mindfulness-based stress reduction in nurses with insomnia: A non-inferiority internet delivered randomized controlled trial | Primary focus is not depression or anxiety |
|  |  |  |
| Gupta et al. (2023) [52] | Effect of cognitive retraining treatment in mild to moderate depressive disorders. | Focus is on another topic |
|  |  |  |
| Gupta et al. (2021) [53] | Feasibility and effectiveness of telecounseling on the psychological problems of frontline healthcare workers amidst COVID-19: A randomized controlled trial from central India. | Not a DMHI |
|  |  |  |
| Hagan et al. (2023) [54] | Gender digital health literacy gap across age: A moderated moderation effect on depression among in-school adolescents in Ghana during COVID-19 | Focus is on another topic |
|  |  |  |
| Hammond et al. (2021) [55] | Psychoeducational social anxiety mobile apps: Systematic search in app stores, content analysis, and evaluation. | Focus is not on LMICs |
|  |  |  |
| Hashmi et al. (2020) [56] | The impact of the COVID-19 pandemic on mental health and service delivery during pregnancy: Role of telepsychiatry. | Does not describe use of a DMHI |
|  |  |  |
| Hatami et al. (2022) [57] | Tele-medicine and improvement of mental health problems in COVID-19 pandemic: A systematic review. | Focus is on mental health in general |
|  |  |  |
| Hazarika et al. (2021) [58] | Profile of distress callers and service utilisation of tele-counselling among the population of Assam, India: An exploratory study during COVID-19 | Not a DMHI |
|  |  |  |
| Hernández-Gómez et al. (2022) [59] | Efficacy of Smartphone Apps in Patients With Depressive Disorders: A Systematic Review. | Focus is global |
|  |  |  |
| Hernani et al. (2023) [60] | Telepsychology as the primary mental health care response to the COVID- 19 pandemic in the Philippines | Does not describe use of a DMHI |
|  |  |  |
| Huckins et al. (2020) [61] | Mental health and behavior of college students during the early phases of the COVID-19 pandemic: Longitudinal smartphone and ecological momentary assessment study | Focus is not on LMICs |
|  |  |  |
| Ibrahim et al. (2024) [62] | Using virtual reality Pablo gaming in the post operative rehabilitation of breast cancer patients: Randomized controlled trial | Primary focus is not depression or anxiety |
|  |  |  |
| Iftikhar et al. (2023) [63] | Virtual reality tourism and technology acceptance: A disability perspective. | Focus is on another topic |
|  |  |  |
| Iqbal et al. (2020) [64] | COVID-19-related issues on tele-counseling helpline in Bangladesh. | Not a DMHI |
|  |  |  |
| Jawed et al. (2021) [65] | Feasibility of a virtual reality intervention in the intensive care unit. | Focus is on another topic |
|  |  |  |
| Jaywant et al. (2023) [66] | Multinational perspectives on changes to psychiatric care during the COVID-19 pandemic: A survey of practicing psychiatrists. | Focus is on another topic |
|  |  |  |
| Jyoti et al. (2024) [67] | An in vivo study to evaluate and compare anxiety and behavior management of pediatric patients using distraction techniques. | Focus is on other topics |
|  |  |  |
| Kalam et al. (2024) [68] | ChatGPT and mental health: Friends or foes? | Focus is on another topic |
|  |  |  |
| Kaynak et al. (2024) [69] | The effect of virtual patient visits in the intensive care unit on postpartum depression. | Focus is not on LMICs |
|  |  |  |
| Khademi et al. (2023) [70] | The effectiveness of telephone-based psychological services to COVID-19. | Not a DMHI |
|  |  |  |
| Khademian et al. (2020) [71] | The effects of mobile apps on stress, anxiety, and depression: Overview of systematic reviews. | Focus is global |
|  |  |  |
| Khaksar et al. (2022) [72] | Reducing maternal stress in pediatric hospitalization during the COVID-19 pandemic by improving family-centered care  with bedside telehealth: A pilot randomized clinical trial. | Primary focus is not depression or anxiety |
|  |  |  |
| Koirala et al. (2021) [73] | The scope of telemedicine in Nepal during COVID-19 pandemic. | Focus is on another topic |
|  |  |  |
| Komariah et al. (2022) [74] | Efficacy of internet-based cognitive behavioral therapy for depression and anxiety among global population during the COVID-19 pandemic: A systematic review and meta-analysis of a randomized controlled trial study. | Focus is global |
|  |  |  |
| Lahoz et al. (2024) [75] | Pediatric conditions and platforms of telemedicine used in Philippine primary care: A cross-sectional study. | Not a DMHI |
|  |  |  |
| Leung et al. (2022) [76] | Sense of coherence mediates the relationship between digital health literacy and anxiety about the future in aging population during the COVID-19 pandemic: A path analysis. | Focus is on another topic |
|  |  |  |
| Liem et al.  (2020) [77] | A digital mental health intervention to reduce depressive symptoms among overseas Filipino workers: protocol for a pilot hybrid type 1 effectiveness- implementation randomized controlled trial. | Focus is not on LMICs |
| Liem et al. (2021)[78] | Digital health applications in mental health care for immigrants and refugees: A rapid review. | Focus is on mental health in general |
|  |  |  |
| Mack et al. (2021) [79] | Mental health and behavior of college students during the COVID-19 pandemic: Longitudinal mobile smartphone and ecological momentary assessment study, part II | Focus is not on LMICs |
|  |  |  |
| Maulik et al. (2020) [80] | The systematic medical appraisal referral and treatment mental health project: Quasi-experimental study to evaluate a technology-enabled mental health services delivery model implemented in rural India. | Not a DMHI |
|  |  |  |
| Meheli et al. (2022) [81] | Understanding people with chronic pain who use  a cognitive behavioral therapy-based artificial intelligence mental health app (Wysa): Mixed methods retrospective observational study. | Focus is global |
|  |  |  |
| Meyer et al. (2021) [82] | User reviews of depression app features: Sentiment  analysis. | Focus is global |
|  |  |  |
| MindKind Consortium (2022) [83] | MindKind: A mixed-methods protocol for the feasibility  of global digital mental health studies in young people. | Focus is global |
|  |  |  |
| Moulaei et al. (2021) [84] | The design and evaluation of a mobile based application to facilitate self-care for pregnant women with preeclampsia during COVID-19 prevalence. | Primary focus is not depression or anxiety |
|  |  |  |
| Mukherjee et al. (2022) [85] | Protocol for process evaluation of SMART mental health cluster randomised control trial: An intervention for management of common mental disorders in India. | Focus is on mental health in general |
|  |  |  |
| Mumtaz et al. (2021) [86] | COVID-19 rehab fright management. | Focus is on another topic |
|  |  |  |
| Najafi et al. (2025) [87] | Tele-exercise in multiple sclerosis: Systematic review and meta-analysis of effects on fatigue, depression, and overall health | Focus is global |
|  |  |  |
| Naslund et al. (2022) [88] | Economic evaluation and costs of telepsychiatry programmes: A systematic review. | Pre-COVID |
|  |  |  |
| Nayak et al. (2020) [89] | Effectiveness of psychological intervention by videoconference for family members with depression of farmers who have committed suicide. | Not a DMHI |
|  |  |  |
| Nazemi et al. (2023) [90] | Tele-mental health during the COVID-19 pandemic: A systematic review of the literature focused on technical aspects and challenges. | Focus is global |
|  |  |  |
| Newman et al. (2024) [91] | Effectiveness of an eHealth intervention for reducing psychological distress and increasing COVID-19 knowledge and protective behaviors among racialized sexual and gender minority adults: A quasi-experimental study (#SafeHandsSafeHearts). | Focus is not on LMICs |
|  |  |  |
| Newson et al. (2022) [92] | Assessment of population well-being with the Mental Health Quotient: Validation study | Focus is on another topic |
|  |  |  |
| Norouzkhani et al. (2024) [93] | Effect of a gamified mobile-based self-management application on disease activity index, quality of life, and mental health in adults with inflammatory bowel disease: A protocol of a randomized controlled trial study. | Focus is on another topic |
|  |  |  |
| Nowrouzi-Kia et al. (2024) [94] | Evaluating the efficacy of telehealth-based treatments for depression in adults: A rapid review and meta-analysis. | Focus is not on LMICs |
|  |  |  |
| Okunogbe et al. (2023) [95] | Utilization of adolescent health services during the  COVID-19 pandemic: evidence on impact and adaptations from a rapid assessment survey in the Philippines. | Does not describe use of a DMHI |
|  |  |  |
| Onu et al. (2024) [96] | Digital psychiatry in Nigeria: A scoping review. | Focus is on mental health in general |
|  |  |  |
| Palacio-Ortiz et al. (2020) [97] | Psychiatric disorders in children and adolescents during the COVID-19 pandemic. | Focus is on mental health in general |
|  |  |  |
| Peralta et al. (2020) [98] | Effectiveness of teleconsultation use in access to mental health services during the coronavirus disease 2019 pandemic in the  Dominican Republic. | Focus is on mental health in general |
|  |  |  |
| Pozuelo et al. (2024) [99] | A narrative-gamified mental health app (Kuamsha) for adolescents in Uganda: Mixed methods feasibility and acceptability study. | Primary focus is not depression or anxiety |
|  |  |  |
| Priyadarsini et al. (2024) [100] | Impact of mindfulness through smartphone applications on the anxiety among B.Sc. nursing students. | Not a DMHI |
|  |  |  |
| Razavi et al. (2020) [101] | Depression screening using mobile phone usage metadata: a machine learning approach. | Focus is not on LMICs |
|  |  |  |
| Rethorst et al. (2024) [102] | Pilot evaluation on an adapted tele-behavioral activation to increase physical activity in persons with depression: a single-arm pilot study. | Focus is not on LMICs |
|  |  |  |
| Rismawan et al. (2021) [103] | Usability, acceptability, and adherence rates of mobile application interventions for prevention or treatment of depression: A systematic review. | Focus is not on LMICs |
|  |  |  |
| Roy et al. (2021) [104] | Mental health implications of COVID-19 pandemic and its response in India. | Focus is on mental health in general |
|  |  |  |
| Ruchi et al. (2023) [105] | Effect of teleyoga before COVID-19 and during pandemic: A narrative review. | Focus is global |
|  |  |  |
| Sarlon et al. (2022) [106] | Effectiveness of a mindfulness-based mobile application for the treatment of depression in ambulatory care: Protocol for a randomized controlled trial. | Focus is not on LMICs |
|  |  |  |
| Savareh et al. (2024) [107] | Pain management in cancer patients: The effectiveness of digital game-based interventions: A rapid literature review | Focus is global |
|  |  |  |
| Scazufca et al. (2022) [108] | A task-shared, collaborative care psychosocial intervention for improving depressive symptomatology among older adults in a socioeconomically deprived area of Brazil (PROACTIVE):  A pragmatic, two-arm, parallel-group, cluster-randomised controlled trial. | Does not describe use of a DHMI |
|  |  |  |
| Shatri et al. (2021) [109] | The role of online psychotherapy in COVID-19: An evidence based clinical review. | Focus is not on LMICs |
|  |  |  |
| Singh et al. (2022) [110] | A study protocol for a randomised controlled trial on the efficacy of yoga as an adjuvant therapy for patients with ankylosing spondylitis amidst COVID-19 pandemic. | Focus is on another topic |
|  |  |  |
| Situmorang (2022) [111] | “When the first session may be the last!": A case report of the implementation of "rapid tele-psychotherapy" with single-session music therapy in the COVID-19 outbreak. | Not a DMHI |
|  |  |  |
| Situmorang (2023) [112] | The efficacy of "rapid tele-psychotherapy" with single-  session music therapy: A personal reflection as a founder. | Focus is on mental health in general |
|  |  |  |
| Sserunkuuma et al. (2023) [113] | Problematic use of the internet, smartphones, and social media among medical students and relationship with depression: An exploratory study | Focus is on another topic |
|  |  |  |
| Stein et al. (2020) [114] | Mental health delivery and neurogenetics discovery in Africa. | Focus is on another topic |
|  |  |  |
| Suarilah et al. (2022) [115] | Effectiveness of telehealth interventions among traumatic brain injury survivors: A systematic review and meta-analysis. | Focus is not on LMICs |
|  |  |  |
| Sultana et al. (2021) [116] | Psychosocial challenges in palliative care: Bridging the gaps using digital health. | Focus is on another topic |
|  |  |  |
| Sutjonong et al. (2023) [117] | Impact of telemedicine on the mental status of parents of children with CHD: A meta-analysis. | Focus is global |
|  |  |  |
| Toni et al. (2024) [118] | An insight into the use of telemedicine technology  for cancer patients during the Covid-19 pandemic: A scoping review. | Focus is on another topic |
|  |  |  |
| Tran et al. (2023) [119] | Psychological impacts of COVID-19 on Vietnamese health workers over the prolonged restricted COVID-19 responses: A cross-sectional study. | Does not describe use of a DMHI |
|  |  |  |
| Tsheten et al. (2023) [120] | Impact of COVID-19 on mental health in Bhutan: A way forward for action. | Does not describe use of a DMHI |
|  |  |  |
| Vo et al. (2023) [121] | Impacts of the COVID-19 pandemic on patients with chronic conditions in Vietnam: A cross-sectional study. | Focus is on another topic |
|  |  |  |
| Wainberg et al. (2021) [122] | Technology and implementation science to forge the future of evidence-based psychotherapies: The PRIDE scale-up study. | Focus is on another topic |
|  |  |  |
| Wani et al. (2024) [123] | Digital mental health interventions for adolescents in low- and middle-income countries: Scoping review | Focus is global |
|  |  |  |
| Yilmaz et al. (2021) [124] | mHealth: Potentials and risks for addressing mental  health and well-being issues among Nepali adolescents. | Focus is on another topic |
|  |  |  |
| Yue et al. (2021) [125] | The relationships between negative emotions and latent classes of smartphone addiction. | Focus is on another topic |
|  |  |  |
| Zandieh et al. (2024) [126] | Therapist-guided remote versus in-person cognitive behavioural therapy: A systematic review and meta-analysis of randomized controlled trials. | Focus is global |
|  |  |  |
| Zhang et al. (2020) [127] | Virtual reality exposure therapy (VRET) for anxiety due to fear of COVID-19 infection: A case series. | Focus is not on LMICs |
| Zhang et al. (2024) [128] | Implementation of a Machine Learning Risk Prediction Model for Postpartum Depression in the Electronic Health Records | Focus is not on LMICs |
|  |  |  |

### References

1. Adiukwu F, Ojeahere M, Adesokun O, Babalola G. Mental health distress during the COVID-19 pandemic in Nigeria: Need for psychological intervention. S Afr J Psychiatr AOSIS (pty) Ltd; 2022;28:1550. PMID:35169505

2. Aga IZ, Khurram SS, Karim M, Muzzamil M, Hashmi S, Shafique K. Employing telepsychiatry services to assess the prevalence and identify mental health disorders using the PHQ-9 and GAD-7 in resource-constrained regions of Dadar Mansehra, Pakistan: an observational cross-sectional study. BMJ Open BMJ Publishing Group; 2023 Dec 10;13(12). PMID:38072482

3. Ahmed MH, Awol SM, Kanfe SG, Hailegebreal S, Debele GR, Dube GN, Guadie HA, Ngusie HS, Klein J. Willingness to use telemedicine during COVID-19 among health professionals in a low income country. Inform Med Unlocked Elsevier Ltd; 2021 Jan 1;27. doi: 10.1016/J.IMU.2021.100783

4. Ahmed MS, Hasan T, Islam S, Ahmed N. Investigating Rhythmicity in App Usage to Predict Depressive Symptoms: Protocol for Personalized Framework Development and Validation Through a Countrywide Study. JMIR Res Protoc JMIR Publications Inc.; 2024 Apr 24;13:e51540. PMID:38657238

5. Ajmera P, Miraj M, Kalra S, Goyal RK, Chorsiya V, Shaik RA, Alzhrani M, Alanazi A, Alqahtani M, Miraj SA, Pawaria S, Mehta V. Impact of telehealth interventions on physiological and psychological outcomes in breast cancer survivors: A meta-analysis of randomised controlled trials. Front Oncol Frontiers Media S.A.; 2023 Jan 5;12. doi: 10.3389/FONC.2022.1017343

6. Akingbade O, Nguyen KT, Chow KM. Effect of mHealth interventions on psychological issues experienced by women undergoing chemotherapy for breast cancer: A systematic review and meta-analysis. J Clin Nurs John Wiley and Sons Inc; 2023 Jul 1;32(13–14):3058–3073. PMID:36168199

7. Alam MZ, Khanam L. Comparison of the young aged and elderly female users’ adoption of mHealth services. Health Care Women Int Taylor and Francis Ltd.; 2022;43(10–11):1259–1283. PMID:35195507

8. Leveraging the Use of mHealth in Patients with Depression among Low- and Middle- Income Countries. Journal of the College of Physicians and Surgeons Pakistan College of Physicians and Surgeons Pakistan; 2020 Apr 1;30(4):458–458. doi: 10.29271/JCPSP.2020.04.458

9. Alqarni AM, Elfaki A, Abdel Wahab M, Aljehani Y, Alkhunaizi AA, Alex J, Othman SA, Amer FH, Alghamdi FA, Alissa KA. Brief tele-mindfulness-based intervention: A multicenter randomized controlled trial. J Family Community Med Wolters Kluwer Medknow Publications; 2023 Jul 1;30(3):180–187. doi: 10.4103/JFCM.JFCM_82_23

10. Andersen NJ, Schwartzman D, Martinez C, Cormier G, Drapeau M. Virtual reality interventions for the treatment of anxiety disorders: A scoping review. J Behav Ther Exp Psychiatry Elsevier Ltd; 2023 Dec 1;81. PMID:36947972

11. Angwenyi V, Fletcher R, Mwangi PM, Kabue M, Odhiambo R, Mulupi S, Obulemire EK, Njoroge E, Ombech E, Mokaya MM, Wesala M, Marangu J, Abubakar A. Engaging fathers(to-be): a pilot study on the adaptation and programme experience  of SMS4baba intervention in Kenya’s informal settlements. BMC Public Health England; 2024 Dec;24(1):3603. PMID:39736598

12. Ansaari N, Rajan SK, Kuruveettissery S. Efficacy of in-person versus digital mental health interventions for postpartum depression: meta-analysis of randomized controlled trials. J Reprod Infant Psychol Routledge; 2024; doi: 10.1080/02646838.2024.2303470

13. Askarizadeh MM, Gholamhosseini L, Khajouei R, Homayee S, Askarizadeh F, Ahmadian L. Determining the impact of mobile-based self-care applications on reducing anxiety  in healthcare providers: a systematic review. BMC Med Inform Decis Mak England; 2025 Jan;25(1):37. PMID:39849432

14. Atiq M, Nazir H, Rahman A, Malik A, Atif N, Surkan PJ. Exploring preference for delivery methods for a Psychosocial Intervention for Prenatal Anxiety: A Qualitative Study from a Tertiary Care Hospital in Pakistan. Cambridge Prisms: Global Mental Health Cambridge University Press (CUP); 2024 May 9;1–28. doi: 10.1017/GMH.2024.59

15. Babaei N, Zamanzadeh V, Pourabbasi M, Avazeh M, Shokoufamanesh A. The effect of virtual reminiscence therapy on depression and anxiety in patients with gastric cancer undergoing chemotherapy. Support Care Cancer Support Care Cancer; 2023 Jan 1;32(1). PMID:38150072

16. Baradwan S, Khadawardi K, Hamid OA, Baradwan A, Alanazi N, Abdelwahed RM, Elshabrawi MN, Elbeltagy ESF, Mohamed SRE, Mohamed SSAE, Abdelhakim AM, Abdelhamed SA. The impact of virtual reality on maternal anxiety, satisfaction, and fetal  outcomes among pregnant women undergoing non-stress tests: A systematic review and meta-analysis of randomized controlled trials. Health Care Women Int England; 2025 Jan;1–16. PMID:39847547

17. Beatty C, Malik T, Meheli S, Sinha C. Evaluating the Therapeutic Alliance With a Free-Text CBT Conversational Agent (Wysa): A Mixed-Methods Study. Front Digit Health Frontiers Media S.A.; 2022 Apr 11;4. doi: 10.3389/FDGTH.2022.847991

18. Ben-Zeev D, Chander A, Tauscher J, Buck B, Nepal S, Campbell A, Doron G. A smartphone intervention for people with serious mental illness: Fully remote randomized controlled trial of CORE. J Med Internet Res JMIR Publications Inc.; 2021 Nov 1;23(11). PMID:34766913

19. Beniwal RP, Sreedaran P, Chari U, Ashok MV, Bhatia T. Protocol of a Multi-centric Randomized Controlled Trial to Evaluate Efficacy of Telephone-Based Psychosocial Interventions on Future Suicide Risk in Suicide Attempters. Indian J Psychol Med SAGE Publications Ltd; 2020 Dec 1;42(6_suppl):S39–S45. doi: 10.1177/0253717620971199

20. Bhardwaj A, Maharjan SM, Magar AJ, Shrestha R, Dongol A, Hagaman A, Van Heerden A, Caracoglia J, Gallo JJ, Murray SM, Kohrt BA. Engaging husbands in a digital mental health intervention to provide tailored counseling for women experiencing postpartum depression: A mixed methods study in Nepal. SSM - Mental Health Elsevier Ltd; 2024 Dec 1;6:100340. doi: 10.1016/j.ssmmh.2024.100340

21. Bhat A, Goud BR, Pradeep JR, Jayaram G, Radhakrishnan R, Srinivasan K. Can Mobile Health Improve Depression Treatment Access and Adherence Among Rural Indian Women? A Qualitative Study. Cult Med Psychiatry Springer; 2020 Dec 1;44(4):461–478. PMID:31916181

22. Bhatia A, Kc M, Gupta L. Increased risk of mental health disorders in patients with RA during the COVID-19 pandemic: a possible surge and solutions. Rheumatol Int Springer Science and Business Media Deutschland GmbH; 2021 May 1;41(5):843–850. PMID:33710419

23. Bikmoradi A, Omidvar S, Roshanaei G, Khatiban M, Harorani M. The impact of telenursing on level of depression, stress and anxiety in discharged patients after coronary artery bypass graft surgery: A randomized clinical trial. J Vasc Nurs J Vasc Nurs; 2023 Sep 1;41(3):89–94. PMID:37684095

24. Bosqui T, McEwen FS, Chehade N, Moghames P, Skavenski S, Murray L, Karam E, Weierstall-Pust R, Pluess M. What drives change in children receiving telephone-delivered Common Elements Treatment Approach (t-CETA)? A multiple n = 1 study with Syrian refugee children and adolescents in Lebanon. Child Abuse Negl Elsevier Ltd; 2023; PMID:37612204

25. Bui LK, Park M, Giap TTT. eHealth interventions for the informal caregivers of people with dementia: A systematic review of systematic reviews. Geriatr Nurs (Minneap) Elsevier Inc.; 2022 Nov 1;48:199–209. PMID:36274510

26. Campisi SC, Ataullahjan A, Baxter JAB, Szatmari P, Bhutta ZA. Mental health interventions in adolescence. Curr Opin Psychol Elsevier B.V.; 2022 Dec 1;48. PMID:36347181

27. Carter H, Araya R, Anjur K, Deng D, Naslund JA. The emergence of digital mental health in low-income and middle-income countries: A review of recent advances and implications for the treatment and prevention of mental disorders. J Psychiatr Res J Psychiatr Res; 2021 Jan 1;133:223–246. PMID:33360867

28. Chakeri A, Jalali E, Ghadi M, Mohamadi M. Evaluating the effect of nurse-led telephone follow-ups (tele-nursing) on the anxiety levels in people with coronavirus. J Family Med Prim Care Medknow; 2020;9(10):5351. doi: 10.4103/JFMPC.JFMPC_847_20

29. Dantes GR, Asril NM, Liem A, Suwastini NKA, Keng SL, Mahayanti NWS. Brief Mobile App-Based Mindfulness Intervention for Indonesian Senior High School Teachers: Protocol for a Pilot Randomized Controlled Trial. JMIR Res Protoc JMIR Res Protoc; 2024;13. PMID:39442169

30. Dergaa I, Fekih-Romdhane F, Hallit S, Loch AA, Glenn JM, Fessi MS, Ben Aissa M, Souissi N, Guelmami N, Swed S, El Omri A, Bragazzi NL, Ben Saad H. ChatGPT is not ready yet for use in providing mental health assessment and interventions. Front Psychiatry Frontiers Media SA; 2023;14. doi: 10.3389/FPSYT.2023.1277756

31. Dikaios E, Sekhon H, Allard A, Vacaflor B, Goodman A, Dwyer E, Lavin-Gonzalez P, Mahdanian A, Park H, Walsh C, Sasi N, Nazar R, Gruber J, Su C-L, Hanganu C, Royal I, Schiavetto A, Cinalioglu K, Rigas C, Launay C, Beauchet O, McDonald E, Seitz D, Kumar S, Nair V, Miresco M, Bruneau M-A, Alexopoulos G, Looper K, Vahia I, Rej S, Bukhari SN. Connecting During COVID-19: A Protocol of a Volunteer-Based Telehealth Program for Supporting Older Adults’ Health. Front Psychiatry Frontiers Media S.A.; 2020 Dec 2;11. doi: 10.3389/fpsyt.2020.598356

32. Dixit S, Nandakumar G. Promoting healthy lifestyles using information technology during the COVID-19  pandemic. Rev Cardiovasc Med Singapore; 2021 Mar;22(1):115–125. PMID:33792253

33. Drissi N, Ouhbi S, Janati Idrissi MA, Ghogho M. An analysis on self-management and treatment-related functionality and  characteristics of highly rated anxiety apps. Int J Med Inform Ireland; 2020 Sep;141:104243. PMID:32768994

34. Dyer J, Wilson K, Badia J, Agot K, Neary J, Njuguna I, Kibugi J, Healy E, Beima-Sofie K, John-Stewart G, Kohler P. The Psychosocial Effects of the COVID-19 Pandemic on Youth Living with HIV in  Western Kenya. AIDS Behav United States; 2021 Jan;25(1):68–72. PMID:32816193

35. Dzinamarira T, Iradukunda PG, Saramba E, Gashema P, Moyo E, Mangezi W, Musuka G. COVID-19 and mental health services in Sub-Saharan Africa: A critical literature  review. Compr Psychiatry United States; 2024 May;131:152465. PMID:38387168

36. El-Refaay SMM, Toivanen-Atilla K, Crego N. Efficacy of technology-based mental health interventions in minimizing mental  health symptoms among in immigrants, asylum seekers or refugees; systematic review. Arch Psychiatr Nurs United States; 2024 Aug;51:38–47. PMID:39034093

37. Escrivá-Martínez T, Vara MD, Czeraniuk N, Denis M, Núñez-Benjumea FJ, Fernández-Luque L, Jiménez-Díaz A, Traver V, Llull JJ, Martínez-Millana A, Garcés-Ferrer J, Miragall M, Herrero R, Enríquez A, Schaefer V, Cervera-Torres S, Villasanti C, Cabral C V., Fernández I, Baños RM. MHealth intervention to improve quality of life in patients with chronic diseases during the COVID-19 crisis in Paraguay: A study protocol for a randomized controlled trial. PLoS One Public Library of Science; 2022 Nov 1;17(11 November). PMID:36346807

38. Farahimanesh S, Serino S, Tuena C, Di Lernia D, Wiederhold BK, Bernardelli L, Riva G, Moradi A. Effectiveness of a Virtual-Reality-Based Self-Help Intervention for Lowering the Psychological Burden during the COVID-19 Pandemic: Results from a Randomized Controlled Trial in Iran. J Clin Med Multidisciplinary Digital Publishing Institute (MDPI); 2023 Mar 1;12(5). doi: 10.3390/JCM12052006

39. Farzandipour M, Sharif R, Anvari S. Effects of mhealth applications on military personnel’s physical and mental health: A systematic review. Mil Psychol Mil Psychol; 2025;37(3):199–207. PMID:38551462

40. Fereidooni M, Toni E, Toni E, Ayatollahi H. Application of virtual reality for supportive care in cancer patients: a  systematic review. Support Care Cancer Germany; 2024 Aug;32(9):570. PMID:39103681

41. Feroz AS, Khoja A, Saleem S. Equipping community health workers with digital tools for pandemic response in  LMICs. Arch Public Health England; 2021 Jan;79(1):1. PMID:33390163

42. Ganesan S, Balasubramanian B, Krishnamurthy P, Govindan R, Mani N. Effects of Tele-Counseling on Reducing Anxiety Levels of COVID-19 Patients in Isolation Wards: An Observational Study. Indian J Psychol Med SAGE Publications Ltd; 2023 Jan 1;45(1):43–46. doi: 10.1177/02537176221139598

43. Garg A, Agrawal R, Velleman R, Rane A, Costa S, Gupta D, Dsouza E, Jambhale A, Sabnis A, Fernandes G, Bhatia U, Nadkarni A. Integrating assisted tele-psychiatry into primary healthcare in Goa, India: a feasibility study. Global Mental Health Cambridge University Press (CUP); 2022;9:26–36. doi: 10.1017/GMH.2021.47

44. Gautam K, Aguilar C, Paudel K, Dhakal M, Wickersham JA, Acharya B, Sapkota S, Deuba K, Shrestha R. Preferences for mHealth Intervention to Address Mental Health Challenges Among Men Who Have Sex With Men in Nepal: Qualitative Study. JMIR Hum Factors JMIR Publications Inc.; 2024 Jan 1;11(1). doi: 10.2196/56002

45. Gautama MSN, Haryani H, Huang TW. Efficacy of smartphone-based virtual reality relaxation in providing comfort to patients with cancer undergoing chemotherapy in oncology outpatient setting in Indonesia: Protocol for a randomised controlled trial. BMJ Open BMJ Publishing Group; 2023 Jul 25;13(7). PMID:37491084

46. Getu MA, Wang P, Addissie A, Seife E, Chen C, Kantelhardt EJ. The effect of cognitive behavioural therapy integrated with activity pacing on cancer-related fatigue, depression and quality of life among patients with breast cancer undergoing chemotherapy in Ethiopia: A randomised clinical trial. Int J Cancer John Wiley and Sons Inc; 2023 Jun 15;152(12):2541–2553. PMID:36744446

47. Gevers-Montoro C, Deldar Z, Furlan A, Lazar EA, Ghalibaf E, Ortega-De Mues A, Khatibi A. From hands-on to remote: Moderators of response to a novel self-management telehealth programme during the COVID-19 pandemic. European Journal of Pain (United Kingdom) John Wiley and Sons Inc; 2022 Jul 1;26(6):1368–1379. PMID:35610958

48. Ghazanfarpour M, Ashrafinia F, Zolala S, Ahmadi A, Jahani Y, Hosseininasab A. Investigating the effectiveness of tele-counseling for the mental health of staff in hospitals and COVID-19 clinics: a clinical control trial. Trends Psychiatry Psychother Sociedade de Psiquiatria do Rio Grande do Sul; 2022;44. PMID:34797967

49. Ghobadi A, Moradpoor H, Sharini H, Khazaie H, Moradpoor P. The effect of virtual reality on reducing patients’ anxiety and pain during  dental implant surgery. BMC Oral Health England; 2024 Feb;24(1):186. PMID:38317209

50. Grande AJ, Ribeiro WS, Faustino C, De Miranda CT, McDaid D, Fry A, De Moraes SHM, De Oliveira SMDVL, De Farias JM, De Tarso Coelho Jardim P, King D, Silva V, Ziebold C, Evans-Lacko S. Effective/cost effective interventions of child mental health problems in low- and middle-income countries (LAMIC): Systematic review. Medicine (United States) Lippincott Williams and Wilkins; 2020 Jan 1;99(1). PMID:31895812

51. Guo W, Nazari N, Sadeghi M. Cognitive-behavioral treatment for insomnia and mindfulness-based stress  reduction in nurses with insomnia: a non-inferiority internet delivered randomized controlled trial. PeerJ United States; 2024;12:e17491. PMID:39071123

52. Gupta A, Kumari S. Effect of cognitive retraining treatment in mild to moderate depressive  disorders. Psicol Reflex Crit Brazil; 2023 Sep;36(1):28. PMID:37721578

53. Gupta S, Kumar M, Rozatkar AR, Basera D, Purwar S, Gautam D, Jahan R. Feasibility and Effectiveness of Telecounseling on the Psychological Problems of Frontline Healthcare Workers Amidst COVID-19: A Randomized Controlled Trial from Central India. Indian J Psychol Med SAGE Publications Ltd; 2021 Jul 1;43(4):343–350. doi: 10.1177/02537176211024537

54. Hagan JE, Quansah F, Frimpong JB, Agormedah EK, Nugba RM, Srem‐Sai M, Schack T. Gender digital health literacy gap across age: A moderated moderation effect on depression among in‐school adolescents in Ghana during COVID‐19. Psychol Sch John Wiley and Sons Inc; 2023 Sep 8;60(9):3452–3468. doi: 10.1002/pits.22942

55. Hammond TE, Lampe L, Campbell A, Perisic S, Brakoulias V. Psychoeducational social anxiety mobile apps: Systematic search in app stores, content analysis, and evaluation. JMIR Mhealth Uhealth JMIR Publications Inc.; 2021 Sep 1;9(9). PMID:34546179

56. Hashmi N, Ullah I, El Hayek S, Shakoor N. The impact of the COVID-19 pandemic on mental health and service delivery during pregnancy: Role of telepsychiatry. Asian J Psychiatr Elsevier B.V.; 2020 Dec 1;54. PMID:33271738

57. Hatami H, Deravi N, Danaei B, Zangiabadian M, Shahidi Bonjar AH, kheradmand A, Nasiri MJ. Tele-medicine and improvement of mental health problems in COVID-19 pandemic: A systematic review. Int J Methods Psychiatr Res John Wiley and Sons Ltd; 2022; PMID:35700080

58. Hazarika M, Das B, Das S, Baruah A, Sharma N, Barua C, Das J, Choudhury S, Hazarika D, Sarma P, Bhandari SS. Profile of distress callers and service utilisation of tele-counselling among the population of Assam, India: an exploratory study during COVID-19. Open J Psychiatry Allied Sci Diva Enterprises Private Limited; 2021;12(1):7. doi: 10.5958/2394-2061.2021.00001.X

59. Hernández-Gómez A, Valdés-Florido MJ, Lahera G, Andrade-González N. Efficacy of Smartphone Apps in Patients With Depressive Disorders: A Systematic Review. Front Psychiatry Frontiers Media S.A.; 2022 Aug 12;13. doi: 10.3389/FPSYT.2022.871966

60. Hernani EV. Telepsychology as the Primary Mental Health Care Response to the COVID-19 Pandemic in the Philippines. Eco-Anxiety and Pandemic Distress Oxford University PressNew York; 2023. p. 162–172. doi: 10.1093/oso/9780197622674.003.0014

61. Huckins JF, daSilva AW, Wang W, Hedlund E, Rogers C, Nepal SK, Wu J, Obuchi M, Murphy EI, Meyer ML, Wagner DD, Holtzheimer PE, Campbell AT. Mental Health and Behavior of College Students During the Early Phases of the COVID-19 Pandemic: Longitudinal Smartphone and Ecological Momentary Assessment Study. J Med Internet Res JMIR Publications Inc.; 2020 Jun 17;22(6):e20185. doi: 10.2196/20185

62. Abdelmoniem Ibrahim A, Aly SM, Youssef ASA, Ragab MMM, Hussein HM. Using Virtual Reality Pablo Gaming in the Post-Operative Rehabilitation of Breast  Cancer Patients: Randomized Controlled Trial. J Clin Med Switzerland; 2024 Dec;13(24). PMID:39768532

63. Iftikhar R, Khan MS, Pasanchay K. Virtual reality tourism and technology acceptance: a disability perspective. Leisure Studies Routledge; 2023 Nov 2;42(6):849–865. doi: 10.1080/02614367.2022.2153903

64. Iqbal Y, Jahan R, Yesmin S, Selim A, Siddique SN. COVID-19-related issues on tele-counseling helpline in Bangladesh. Asia-Pacific Psychiatry John Wiley and Sons Inc; 2021 Jun 1;13(2). PMID:32808472

65. Jawed YT, Golovyan D, Lopez D, Khan SH, Wang S, Freund C, Imran S, Hameed U Bin, Smith JP, Kok L, Khan BA. Feasibility of a virtual reality intervention in the intensive care unit. Heart and Lung Mosby Inc.; 2021 Nov 1;50(6):748–753. PMID:34217986

66. Jaywant A, Aulitzky W, Avari J, Buchheim A, Dubin M, Galffy M, Khoodoruth MAS, Maytal G, Skelin M, Sperner-Unterweger B, Barnhill JW, Fleischhacker WW. Multinational perspectives on changes to psychiatric care during the COVID-19 pandemic: a survey of practicing psychiatrists. Neuropsychiatrie Springer; 2023 Sep 1;37(3):115–121. PMID:36600105

67. Jyoti D, Shams SA, Anand P, Sagar S, Raj N, Singh S. An In Vivo Study to Evaluate and Compare Anxiety and Behavior Management of  Pediatric Patients Using Distraction Techniques. J Pharm Bioallied Sci India; 2024 Jul;16(Suppl 3):S2116–S2118. PMID:39346360

68. Kalam KT, Rahman JM, Islam MR, Dewan SMR. ChatGPT and mental health: Friends or foes? Health Sci Rep John Wiley and Sons Inc; 2024 Feb 1;7(2). doi: 10.1002/HSR2.1912

69. Kaynak S, Yılmaz HB, Çağlar A, Özdil M. The Effect of Virtual Patient Visits in the Intensive Care Unit on Postpartum  Depression. Iran J Public Health Iran; 2024 Aug;53(8):1777–1784. PMID:39415864

70. Khademi M, Vaziri-Harami R, Mashadi AM, Seif P, Babazadehdezfoly A. The Effectiveness of Telephone-based Psychological Services to COVID-19. Clin Pract Epidemiol Ment Health United Arab Emirates; 2023;19:e174501792307270. PMID:37916207

71. Khademian F, Aslani A, Bastani P. The effects of mobile apps on stress, anxiety, and depression: overview of systematic reviews. Int J Technol Assess Health Care Int J Technol Assess Health Care; 2020;37. PMID:33314997

72. Khaksar S, Maroufi M, Kalhor F. Reducing Maternal Stress in Pediatric Hospitalization during the COVID-19 Pandemic by Improving Family-Centered Care with Bedside Telehealth: A Pilot Randomized Clinical Trial. Iran J Psychiatry Tehran University of Medical Sciences; 2022 Jan 1;17(4):361–368. doi: 10.18502/IJPS.V17I4.10684

73. Koirala S, Parajuli BR. The scope of telemedicine in nepal during covid-19 pandemic. Journal of the Nepal Medical Association Nepal Medical Association; 2021 Dec 11;59(244):1326–1327. PMID:35199788

74. Komariah M, Amirah S, Faisal EG, Prayogo SA, Maulana S, Platini H, Suryani S, Yosep I, Arifin H. Efficacy of Internet-Based Cognitive Behavioral Therapy for Depression and Anxiety among Global Population during the COVID-19 Pandemic: A Systematic Review and Meta-Analysis of a Randomized Controlled Trial Study. Healthcare (Switzerland) MDPI; 2022 Jul 1;10(7). doi: 10.3390/HEALTHCARE10071224

75. Lahoz ACF, Dans LF, Tan-Lim CSC, Tomas AC V, Galingana CLT, Sanchez JT, Aquino MRN, Amit AML, Rey MP, Panganiban JMS, Dans AL. Pediatric Conditions and Platforms of Telemedicine Used in Philippine Primary  Care: A Cross-sectional Study. Acta Med Philipp Philippines; 2024;58(15):39–45. PMID:39308879

76. Leung AYM, Parial LL, Tolabing MC, Sim T, Mo P, Okan O, Dadaczynski K. Sense of coherence mediates the relationship between digital health literacy and anxiety about the future in aging population during the COVID-19 pandemic: a path analysis. Aging Ment Health Routledge; 2022;26(3):544–553. PMID:33438448

77. Liem A, Garabiles MR, Pakingan KA, Chen W, Lam AIF, Burchert S, Hall BJ. A digital mental health intervention to reduce depressive symptoms among overseas Filipino workers: protocol for a pilot hybrid type 1 effectiveness-implementation randomized controlled trial. Implement Sci Commun BioMed Central Ltd; 2020 Dec 1;1(1). doi: 10.1186/S43058-020-00072-Y

78. Liem A, Natari RB, Jimmy, Hall BJ. Digital Health Applications in Mental Health Care for Immigrants and Refugees: A Rapid Review. Telemedicine and e-Health Mary Ann Liebert Inc.; 2021 Jan 1;27(1):3–16. PMID:32498658

79. Mack DL, DaSilva AW, Rogers C, Hedlund E, Murphy EI, Vojdanovski V, Plomp J, Wang W, Nepal SK, Holtzheimer PE, Wagner DD, Jacobson NC, Meyer ML, Campbell AT, Huckins JF. Mental health and behavior of college students during the covid-19 pandemic: Longitudinal mobile smartphone and ecological momentary assessment study, part II. J Med Internet Res JMIR Publications Inc.; 2021 Jun 1;23(6). PMID:33900935

80. Maulik PK, Devarapalli S, Kallakuri S, Bhattacharya A, Peiris D, Patel A. The systematic medical appraisal referral and treatment mental health project: Quasi-experimental study to evaluate a technology-enabled mental health services delivery model implemented in Rural India. J Med Internet Res JMIR Publications Inc.; 2020 Feb 1;22(2). PMID:32130125

81. Meheli S, Sinha C, Kadaba M. Understanding People With Chronic Pain Who Use a Cognitive Behavioral Therapy-Based Artificial Intelligence Mental Health App (Wysa): Mixed Methods Retrospective Observational Study. JMIR Hum Factors JMIR Publications Inc.; 2022 Apr 1;9(2). doi: 10.2196/35671

82. Meyer J, Okuboyejo S. User Reviews of Depression App Features: Sentiment Analysis. JMIR Form Res JMIR Publications Inc.; 2021 Dec 1;5(12). doi: 10.2196/17062

83. Adeyemi FO, Areán PA, Bampton E, Bradic L, Burn AM, Carey E, Carlson SP, Collins PY, Concepcion T, Damji M, Doerr M, Dunbar JC, Fazel M, Fernandes B, Finchilescu G, Ford TJ, Freeman M, Fillipo IRG, Hodgson J, Kalha J, Karani M, Kellen MR, Kemp CG, Lindani S, Mangravite LM, Marten C, Mata-Greve F, Moore E, Mounts E, Neelakantan L, Omberg L, Pasquale L, Pathare S, Ranganathan S, Sams N, Scanlan E, Shah H, Short S, Sibisi R, Sieberts SK, Simon S, Sumant S, Suver C, Van Vught C, Velloza J, Zingela Z. MindKind: A mixed-methods protocol for the feasibility of global digital mental health studies in young people. Wellcome Open Res F1000 Research Ltd; 2022;6. doi: 10.12688/WELLCOMEOPENRES.17167.2

84. Moulaei K, Bahaadinbeigy K, Ghaffaripour Z, Ghaemi MM. The design and evaluation of a mobile based application to facilitate self-care for pregnant women with preeclampsia during covid-19 prevalence. J Biomed Phys Eng Shriaz University of Medical Sciences; 2021 Aug 1;11(4):551–560. doi: 10.31661/JBPE.V0I0.2103-1294

85. Mukherjee A, Daniel M, Kallakuri S, Kaur A, Devarapalli S, Raman U, Thornicroft G, Essue BM, Praveen D, Sagar R, Kant S, Saxena S, Patel A, Peiris D, Maulik PK. Protocol for process evaluation of SMART Mental Health cluster randomised control trial: an intervention for management of common mental disorders in India. BMJ Open BMJ Publishing Group; 2022 Jun 17;12(6). PMID:35715180

86. Mumtaz N, Saqulain G, Mumtaz N. COVID-19 Rehab Fright Management. Pak J Med Sci Pakistan; 2021;37(1):277–282. PMID:33437291

87. Najafi P, Motl RW, Moghadasi M. Tele-exercise in multiple sclerosis: Systematic review and meta-analysis of  effects on fatigue, depression, and overall health. Mult Scler Relat Disord Netherlands; 2025 Jan;93:106225. PMID:39709696

88. Naslund JA, Mitchell LM, Joshi U, Nagda D, Lu C. Economic evaluation and costs of telepsychiatry programmes: A systematic review. J Telemed Telecare SAGE Publications Ltd; 2022 Jun 1;28(5):311–330. PMID:32746762

89. Nayak RB, Bhatia T, Mahadevaiah M, Bheemappa A. Effectiveness of Psychological Intervention by Videoconference for Family Members with Depression of Farmers Who Have Committed Suicide. Indian J Psychol Med SAGE Publications Ltd; 2020 Dec 1;42(6_suppl):S46–S50. PMID:33487802

90. Nazemi M, Kiani S, Zakerabasali S. Tele-mental health during the COVID-19 pandemic: A systematic review of the literature focused on technical aspects and challenges. Health Sci Rep John Wiley and Sons Inc; 2023 Jan 1;6(10). doi: 10.1002/HSR2.1637

91. Newman PA, Chakrapani V, Massaquoi N, Williams CC, Tharao W, Tepjan S, Roungprakhon S, Forbes J, Sebastian S, Akkakanjanasupar P, Aden M. Effectiveness of an eHealth intervention for reducing psychological distress and increasing COVID-19 knowledge and protective behaviors among racialized sexual and gender minority adults: A quasi-experimental study (#SafeHandsSafeHearts). PLoS One Public Library of Science; 2024 May 1;19(5 May). PMID:38701074

92. Newson JJ, Pastukh V, Thiagarajan TC. Assessment of Population Well-being With the Mental Health Quotient: Validation Study. JMIR Ment Health JMIR Publications Inc.; 2022 Apr 1;9(4). doi: 10.2196/34105

93. Norouzkhani N, Faramarzi M, Bahari A, Shokri Shirvani J, Shirvani YE, Eslami S, Tabesh H. Effect of a gamified mobile-based self-management application on disease activity index, quality of life, and mental health in adults with inflammatory bowel disease: A protocol of a randomized controlled trial study. Health Sci Rep John Wiley and Sons Inc; 2024 May 1;7(5). doi: 10.1002/HSR2.2109

94. Nowrouzi-Kia B, Bani-Fatemi A, Jackson TD, Li AKC, Chattu VK, Lytvyak E, Deibert D, Dennett L, Ferguson-Pell M, Hagtvedt R, Els C, Durand-Moreau Q, Gross DP, Straube S. Evaluating the Efficacy of Telehealth-Based Treatments for Depression in Adults:  A Rapid Review and Meta-Analysis. J Occup Rehabil Netherlands; 2024 Nov; PMID:39485666

95. Okunogbe A, Meekins M, Saalim K, Conti-Lopez MA, Benabaye RM, Mendoza OM, Julio R, Stan L, Bisson C. Utilization of adolescent health services during the COVID-19 pandemic: evidence on impact and adaptations from a rapid assessment survey in the Philippines. BMC Public Health BioMed Central Ltd; 2023 Dec 1;23(1). PMID:36918863

96. Onu JU, Onyeka TC. Digital psychiatry in Nigeria: A scoping review. South African Journal of Psychiatry AOSIS (pty) Ltd; 2024;30. doi: 10.4102/SAJPSYCHIATRY.V30I0.2115

97. Palacio-Ortiz JD, Londoño-Herrera JP, Nanclares-Márquez A, Robledo-Rengifo P, Quintero-Cadavid CP. Psychiatric disorders in children and adolescents during the COVID-19 pandemic. Rev Colomb Psiquiatr Elsevier Doyma; 2020 Oct 1;49(4):279–288. PMID:33328021

98. Peralta EA, Taveras M. Effectiveness of teleconsultation use in access to mental health services during the coronavirus disease 2019 pandemic in the Dominican Republic. Indian J Psychiatry Wolters Kluwer Medknow Publications; 2020 Sep 1;62(9):S492–S494. doi: 10.4103/PSYCHIATRY.INDIANJPSYCHIATRY_1047_20

99. R Pozuelo J, Nabulumba C, Sikoti D, Davis M, Gumikiriza-Onoria JL, Kinyanda E, Moffett B, van Heerden A, O’Mahen HA, Craske M, Sulaiman M, Stein A. A Narrative-Gamified Mental Health App (Kuamsha) for Adolescents in Uganda: Mixed  Methods Feasibility and Acceptability Study. JMIR Serious Games Canada; 2024 Dec;12:e59381. PMID:39700489

100. Priyadarsini A, Sangavi H, Jagadeeswari J, Cecyli C, Dayana B. Impact of Mindfulness through Smartphone Applications on the Anxiety among B.Sc.  Nursing Students. J Pharm Bioallied Sci India; 2024 Jul;16(Suppl 3):S2904–S2906. PMID:39346392

101. Razavi R, Gharipour A, Gharipour M. Depression screening using mobile phone usage metadata: a machine learning approach. Journal of the American Medical Informatics Association Oxford University Press; 2020 Apr 1;27(4):522–530. PMID:31977041

102. Rethorst CD, Trombello JM, Chen PM, Carmody TJ, Goodman LC, Lazalde A, Trivedi MH. Pilot evaluation on an adapted tele-behavioral activation to increase physical  activity in persons with depression: a single-arm pilot study. BMC Psychol England; 2024 Nov;12(1):643. PMID:39522018

103. Rismawan W, Marchira CR, Rahmat I. Usability, acceptability, and adherence rates of mobile application interventions for prevention or treatment of depression: A systematic review. J Psychosoc Nurs Ment Health Serv Slack Incorporated; 2021 Feb 1;59(2):41–47. PMID:33180949

104. Roy A, Singh AK, Mishra S, Chinnadurai A, Mitra A, Bakshi O. Mental health implications of COVID-19 pandemic and its response in India. International Journal of Social Psychiatry SAGE Publications Ltd; 2021 Aug 1;67(5):587–600. PMID:32873106

105. Ruchi D, Saloni M, Ramesh K, Indrajeet S, Sunil K, Anissa MA, Girish S. Effect of Teleyoga before COVID-19 and during Pandemic: A Narrative Review. Recent Advances in Anti-Infective Drug Discovery Bentham Science Publishers; 2023;18(3):178–187. PMID:36815650

106. Sarlon J, Doll JPK, Schmassmann A, Brand S, Ferreira N, Muehlhauser M, Urech-Meyer S, Schweinfurth N, Lang UE, Bruehl AB. Effectiveness of a Mindfulness-Based Mobile Application for the Treatment of Depression in Ambulatory Care: Protocol for a Randomized Controlled Trial. JMIR Res Protoc JMIR Publications Inc.; 2022 Mar 1;11(3). doi: 10.2196/33423

107. Savareh BA, Karandish Z, Farhoudi F, Bashiri A. Pain Management in Cancer Patients: The Effectiveness of Digital Game-based  Interventions: A Rapid Literature Review. Healthc Inform Res Korea (South); 2024 Oct;30(4):297–311. PMID:39551917

108. Scazufca M, Nakamura CA, Seward N, Moreno-Agostino D, van de Ven P, Hollingworth W, Peters TJ, Araya R. A task-shared, collaborative care psychosocial intervention for improving depressive symptomatology among older adults in a socioeconomically deprived area of Brazil (PROACTIVE): a pragmatic, two-arm, parallel-group, cluster-randomised controlled trial. Lancet Healthy Longev Elsevier Ltd; 2022 Oct 1;3(10):e690–e702. doi: 10.1016/S2666-7568(22)00194-5

109. Shatri H, Prabu OG, Tetrasiwi EN, Faisal E, Putranto R, Ismail RI. The Role of Online Psychotherapy in COVID-19: An Evidence Based Clinical Review. Acta Med Indones Indonesia; 2021 Jul;53(3):352–359. PMID:34611077

110. Singh J, Jha M, Metri K, Mohanty S, Singh A, Tekur P. A study protocol for a randomised controlled trial on the efficacy of yoga as an adjuvant therapy for patients with Ankylosing spondylitis amidst COVID-19 pandemic. Adv Integr Med Elsevier Australia; 2022 Mar 1;9(1):75–79. doi: 10.1016/J.AIMED.2021.11.001

111. Situmorang DDB. “When the first session may be the last!”: A case report of the implementation of “rapid tele-psychotherapy” with single-session music therapy in the COVID-19 outbreak. Palliat Support Care Palliat Support Care; 2022 Apr 4;20(2):290–295. PMID:34399867

112. Situmorang DDB. The efficacy of “rapid tele-psychotherapy” with single-session music therapy: A personal reflection as a founder. Palliat Support Care Cambridge University Press; 2022; PMID:35317880

113. Sserunkuuma J, Kaggwa MM, Muwanguzi M, Najjuka SM, Murungi N, Kajjimu J, Mulungi J, Kihumuro RB, Mamun MA, Griffiths MD, Ashaba S. Problematic use of the internet, smartphones, and social media among medical students and relationship with depression: An exploratory study. Ballarotto G, editor. PLoS One Public Library of Science; 2023 May 26;18(5):e0286424. doi: 10.1371/journal.pone.0286424

114. Stein DJ, Lehner T, Lombard Z, Pringle B, Senthil G, Uddin M. Mental health delivery and neurogenetics discovery in Africa. Lancet Psychiatry Elsevier Ltd; 2020 Jun 1;7(6):473–474. PMID:32445676

115. Suarilah I, Zulkarnain H, Saragih ID, Lee BO. Effectiveness of telehealth interventions among traumatic brain injury survivors: A systematic review and meta-analysis. J Telemed Telecare SAGE Publications Ltd; 2022; doi: 10.1177/1357633X221102264

116. Sultana A, Tasnim S, Sharma R, Pawar P, Bhattcharya S, Hossain MM. Psychosocial challenges in palliative care: Bridging the gaps using digital health. Indian J Palliat Care Scientific Scholar; 2021 Jul 1;27(3):442–447. doi: 10.25259/IJPC_381_20

117. Sutjonong T, Kalangi OAH, Pulinggomang BB, Husada D. Impact of telemedicine on the mental status of parents of children with CHD: A meta-analysis. Pediatrics International John Wiley and Sons Inc; 2023 Jan 1;65(1). PMID:36562667

118. Toni E, Ayatollahi H. An insight into the use of telemedicine technology for cancer patients during the Covid-19 pandemic: a scoping review. BMC Med Inform Decis Mak BioMed Central Ltd; 2024 Dec 1;24(1). PMID:38641567

119. Tran B, Nguyen MT, Auquier P, Boyer L, Fond G, Vu GT, Hoang TP, Ho PT, Nguyen TH, Latkin CA, Ho CSH, Ho RCM, Zhang MWB. Psychological impacts of COVID-19 on Vietnamese health workers over the prolonged restricted COVID-19 responses: a cross-sectional study. BMJ Open BMJ Publishing Group; 2023 Aug 3;13(8). PMID:37536968

120. Tsheten T, Chateau D, Dorji N, Pokhrel HP, Clements ACA, Gray DJ, Wangdi K. Impact of COVID-19 on mental health in Bhutan: a way forward for action. The Lancet regional health Southeast Asia England; 2023 Apr;11:100179. PMID:37020787

121. Vo TH, Nguyen TH, Nguyen HC, Nguyen TH. Impacts of the COVID-19 pandemic on patients with chronic conditions in Vietnam: A cross-sectional study. Chronic Illn SAGE Publications Ltd; 2023; PMID:37448152

122. Wainberg ML, Gouveia ML, Stockton MA, Feliciano P, Suleman A, Mootz JJ, Mello M, Fiks Salem A, Greene MC, Bezuidenhout C, Ngwepe P, Lovero KL, Fortunato Dos Santos P, Schriger SH, Mandell DS, Mulumba R, Neves Anube A, Mabunda D, Mandlate F, Cournos F, Alves-Bradford JM, Nicholson T, Kann B, Fumo W, Duarte CS, De Jesus Mari J, Mello MF, Mocumbi AO, Oquendo MA, Weissman MM. Technology and implementation science to forge the future of evidence-based psychotherapies: The PRIDE scale-up study. Evid Based Ment Health BMJ Publishing Group; 2021 Feb 1;24(1):19–24. PMID:33177149

123. Wani C, McCann L, Lennon M, Radu C. Digital Mental Health Interventions for Adolescents in Low- and Middle-Income  Countries: Scoping Review. J Med Internet Res Canada; 2024 Oct;26:e51376. PMID:39471371

124. Yilmaz SK, Bohara AK. mHealth: Potentials and Risks for Addressing Mental Health and Well-Being Issues Among Nepali Adolescents. Front Public Health Frontiers Media S.A.; 2021 Apr 23;9. PMID:33968868

125. Yue H, Zhang X, Sun J, Liu M, Li C, Bao H. The relationships between negative emotions and latent classes of smartphone  addiction. PLoS One United States; 2021;16(3):e0248555. PMID:33720952

126. Zandieh S, Abdollahzadeh SM, Sadeghirad B, Wang L, McCabe RE, Yao L, Inness BE, Pathak A, Couban RJ, Crandon H, Torabiardakani K, Bieling P, Busse JW. Therapist-guided remote versus in-person cognitive behavioural therapy: a systematic review and meta-analysis of randomized controlled trials. CMAJ Canadian Medical Association Journal Canadian Medical Association; 2024 Mar 18;196(10):E327–E340. PMID:38499303

127. Zhang W, Paudel D, Shi R, Liang J, Liu J, Zeng X, Zhou Y, Zhang B. Virtual reality exposure therapy (Vret) for anxiety due to fear of covid-19 infection: A case series. Neuropsychiatr Dis Treat Dove Medical Press Ltd; 2020;16:2669–2675. PMID:33192065

128. Zhang Y, Joly R, Beecy AN, Principe S, Satpathy S, Gore A, Reilly T, Lang M, Sathi N, Uy C, Adams M, Israel M. Implementation of a Machine Learning Risk Prediction Model for Postpartum  Depression in the Electronic Health Records. AMIA Jt Summits Transl Sci Proc United States; 2024;2024:1057–1066. PMID:39444417
